# Climate Exposures Increase the Risk of Anemia Among Reproductive‐Age Women in Low‐ and Middle‐Income Countries

**DOI:** 10.1002/ajhb.70297

**Published:** 2026-08-31

**Authors:** Alfredo J. Rojas, Clark Gray, Brian C. Thiede, Amanda L. Thompson

**Affiliations:** ^1^ Biobehavioral Health Department Pennsylvania State University University Park Pennsylvania USA; ^2^ Department of Geography and Environment University of North Carolina at Chapel Hill Chapel Hill North Carolina USA; ^3^ Department of Agricultural Economics, Sociology, and Education Pennsylvania State University University Park Pennsylvania USA; ^4^ Department of Anthropology University of North Carolina at Chapel Hill Chapel Hill North Carolina USA

**Keywords:** anemia, climate, DHS, health

## Abstract

Climate change is having significant demographic and health impacts on human populations around the world. There is growing empirical evidence about the impacts of climate variability and change on population health, but this work has focused disproportionately on children and aggregate indicators of weight and (or) height. We extend this literature by focusing on a more targeted indicator of health—anemia—among reproductive‐age women. To do so, we combine Demographic and Health Surveys data for approximately 1.3 million women (ages 15–49 years) with climate and geographic data to analyze climate's impact on anemia risk. We find that unusually hot and wet conditions increase anemia and that exposure to climate extremes—when temperature and precipitation are well above or below average—has even stronger associations with anemia. Specifically, hot conditions as well as hot‐and‐dry and hot‐and‐wet conditions increase the risk of anemia, underlining the importance of temperature as well as combinations of environmental exposures for human health and wellbeing.

## 1 Introduction and Background

Climate change is affecting human populations around the world, influencing where people live, their living standards, and their health (Garip and Reed 2025; Grace et al. 2025). While the health and nutritional impacts of climate have been increasingly studied over recent years, research in this area has focused disproportionately on children and on anthropometric indicators such as weight and (or) height (Andriano 2023; Blom et al. 2022; Dimitrova and Muttarak 2020). Less attention has been given to more granular indicators of health or nutrition (see, e.g., Kumar et al. 2016; Zhu et al. 2023 for an exception), which may be plausibly affected by environmental conditions and their downstream effects but are not well‐captured by measures of height or weight. Measuring these impacts is necessary to fully understand the health impacts of climate change and quantify their social costs.

The presence of anemia is one such indicator. Anemia is a disorder in which blood cannot adequately transport oxygen around the body. The incidence of anemia is an important health issue with far‐reaching demographic and socioeconomic consequences since it can cause debilitating symptoms (i.e., fatigue and weakness), reduce productivity, and increase mortality (WHO 2023). It is also associated with adverse reproductive outcomes, including increased risks for health complications and mortality among mothers and stillbirth as well as preterm birth among children (Beckert et al. 2019; Kinyoki et al. 2021). The disorder is a major public health challenge around the world, especially in low‐ and middle‐income countries (LMICs). Indeed, estimates suggest that more than 3 in 10 reproductive‐age women (31.6% in 2018) in LMICs are anemic (Kinyoki et al. 2021, 1762). Within subgroups, the prevalence in non‐pregnant women decreased only modestly from 31% in 2000 to 30% in 2019; the prevalence in pregnant women, on the other hand, decreased from 41% to 36% from 2000 to 2019 (Stevens et al. 2022).

Efforts to reduce anemia rates are underway, but global progress is slow and uneven across world regions (Mason et al. 2013). While the barriers to such progress are multiple and multi‐dimensional, there are plausible reasons to think that environmental variability associated with global climate change may undermine efforts to reduce anemia. Climatic conditions can potentially increase anemia risk through direct and indirect impacts on food security and disease prevalence, but few studies have examined these relationships empirically. We address that question here by exploring the impact of climate anomalies on anemia in women of reproductive age (ages 15–49). We compile and analyze a global sample of approximately 1.3 million women from nationally representative surveys for 46 countries. Specifically, we use data from the Demographic and Health Survey (DHS) Program, the Climatic Research Unit Time Series (CRU TS), and global administrative boundaries to estimate the impact of temperature and precipitation anomalies on women's anemia status. Building on recent studies, we hypothesize that positive temperature and precipitation anomalies (unusually hot and wet conditions) will increase anemia risk and, to preview our results, find evidence of such impacts. With this in mind, we proceed by outlining our conceptual framework next, followed by a description of our empirical strategy. We then present the results, and we conclude by discussing the broader implications of our findings and tasks for future research to address.

### 1.1 Climatic Dimensions of Health and Anemia Risk

This study contributes to the growing body of work on the climatic dimensions of health outcomes. Climate change may lead to worse health outcomes around the world, especially in LMICs (IPCC 2023). In these regions, which are already disproportionately impacted by anemia, climate change may alter the frequency of drought and floods, decrease food production, and increase infectious disease transmission (Dimitrova et al. 2023; Levy and Patz 2015; WHO 2023). Currently, social scientists are exploring the impacts of climate exposures on social and health outcomes like stunting and wasting in children, underweight among adults, and reproductive health in women (Grace 2017; Mueller and Gray 2018; Thiede and Gray 2020). Conditions like extreme heat, drought, and extreme precipitation may lead to worse health outcomes (Carleton and Hsiang 2016). These outcomes can include exposure to deadly heat events, diarrheal infections among children, and infant mortality (Dimitrova et al. 2023; Geruso and Spears 2018; Mora et al. 2017). The present analysis builds on this previous literature by focusing on anemia, an important measure of food insecurity and health, in adult women in vulnerable regions around the world.

The major proximate causes of anemia are deficiencies in key nutrients such as iron, infectious diseases, inflammation, and genetic disorders (Balarajan et al. 2011; Chaparro and Suchdev 2019). Climate can plausibly influence anemia through two of these pathways, food and nutritional security and disease. First, climate change can potentially affect food security and contribute to malnutrition by reducing agricultural yields, reducing labor productivity, and destabilizing food prices (Fischer et al. 2014; Ghose et al. 2016; Ray et al. 2019; Wheeler and von Braun 2013; Carleton and Hsiang 2016). Specifically, climate conditions like droughts, cyclones, and floods may increase anemia risk due to their impact on food security (Sindayihebura and Nkunzimana 2020; Win and Ko 2018). Food insecurity can lead to anemia through micronutrient deficiencies, changes in food availability or type, and increased prices for healthy foods. Furthermore, anemia risk increases when nutritional demands increase during adolescence, pregnancy, and motherhood (Bellizzi et al. 2021; Gebremedhin and Enquselassie 2011; Hallberg 2001; Sunuwar et al. 2020; WHO 2023). Women in LMICs, a subpopulation already at risk, may develop anemia if they cannot consume enough iron or other micronutrients needed for iron absorption (Moradi et al. 2018; Sorensen, Murray, et al. 2018). While iron deficiency is one of the most common causes of anemia, deficiencies in other nutrients such as vitamin A and B vitamins can result in anemia since they are crucial for iron metabolism and healthy hemoglobin levels.

Second, the spread of pathogens is influenced by climate change (Altizer et al. 2013). Hookworms, malaria, and schistosomiases are diseases that contribute to anemia (Chaparro and Suchdev 2019; Correa‐Agudelo et al. 2021), and exposures can be altered via floods, droughts, rainfall patterns, and other climate‐related processes (Mora et al. 2022). In the case of malaria, parasites require iron for growth, and it can lead to anemia in humans by disrupting iron metabolism in various ways. One way is through the destruction of parasitized and non‐parasitized red blood cells. Moreover, temperature, precipitation, and context‐specific ecological factors contribute to the development of the malaria parasite (Stresman 2010). Climate change may cause changes in the distribution of malaria in certain world regions. For example, warming may promote malaria expansion toward the East African highlands while other regions may become less suitable for mosquito vectors (Caminade et al. 2019).

Other ways climate can influence nutrition are not directly related to precipitation or temperature. For example, increasing carbon dioxide levels in the atmosphere may decrease important nutrients in crops, such as zinc and iron, potentially worsening nutrient deficiencies across vulnerable world regions (Smith et al. 2017). Climate processes, therefore, contribute to the underlying causes of anemia in populations already at risk of exposure.

Scholars have analyzed the ways health, nutritional, and sociodemographic factors influence anemia. For example, Body Mass Index (BMI), an important measure of nutritional status, is a risk factor for anemia identified across world regions. Low BMI or underweight may contribute to anemia risk while higher BMI levels may reduce the risk (Hakizimana et al. 2019; Sunuwar et al. 2020; Teshome et al. 2022; Win and Ko 2018; Worku et al. 2022). This pattern is not always straightforward, as women with high BMI values can also be at increased risk of low iron (Cepeda‐Lopez et al. 2011; Jones et al. 2017). Scholars have also identified other important determinants of anemia that help inform this analysis, such as low wealth status, lower education status, type of residence, currently breastfeeding, using certain contraceptives, ever terminated pregnancy, marital status, toilet facility, and source of drinking water as noted in various studies (Alem et al. 2023; Gautam et al. 2019; Harding et al. 2018; Teshale et al. 2020; Teshome et al. 2022). While anemia is a challenge in many LMICs, subpopulation variation exists such as among the Shuar in Ecuador who have lower rates of anemia compared to other populations in South America (DeLouize et al. 2022). Smaller scale studies can provide valuable insights into the unique factors that contribute to anemia across subpopulations.

### 1.2 Objectives

The overall goal of this study is to measure the associations between exposures to temperature and precipitation anomalies and anemia risk among reproductive‐aged women across LMICs globally. Toward this broad goal, we begin, first, by measuring the overall association between environmental exposures and anemia among women across our sample, using a range of exposure measures that account for non‐linearities in and interactions between temperature and precipitation exposures. Second, we assess whether the links between climate anomalies and anemia risk vary across major sub‐populations in our sample, including by world region, between rural and urban areas, by women's pregnancy status, and education. Third and finally, we conduct parallel analyses of climate effects on women's underweight (low body mass index (BMI)) as a means of exploring whether any detected impacts on anemia are likely to be operating through a nutritional pathway.

## 2 Data and Methods

### 2.1 Data

**FIGURE 1:**
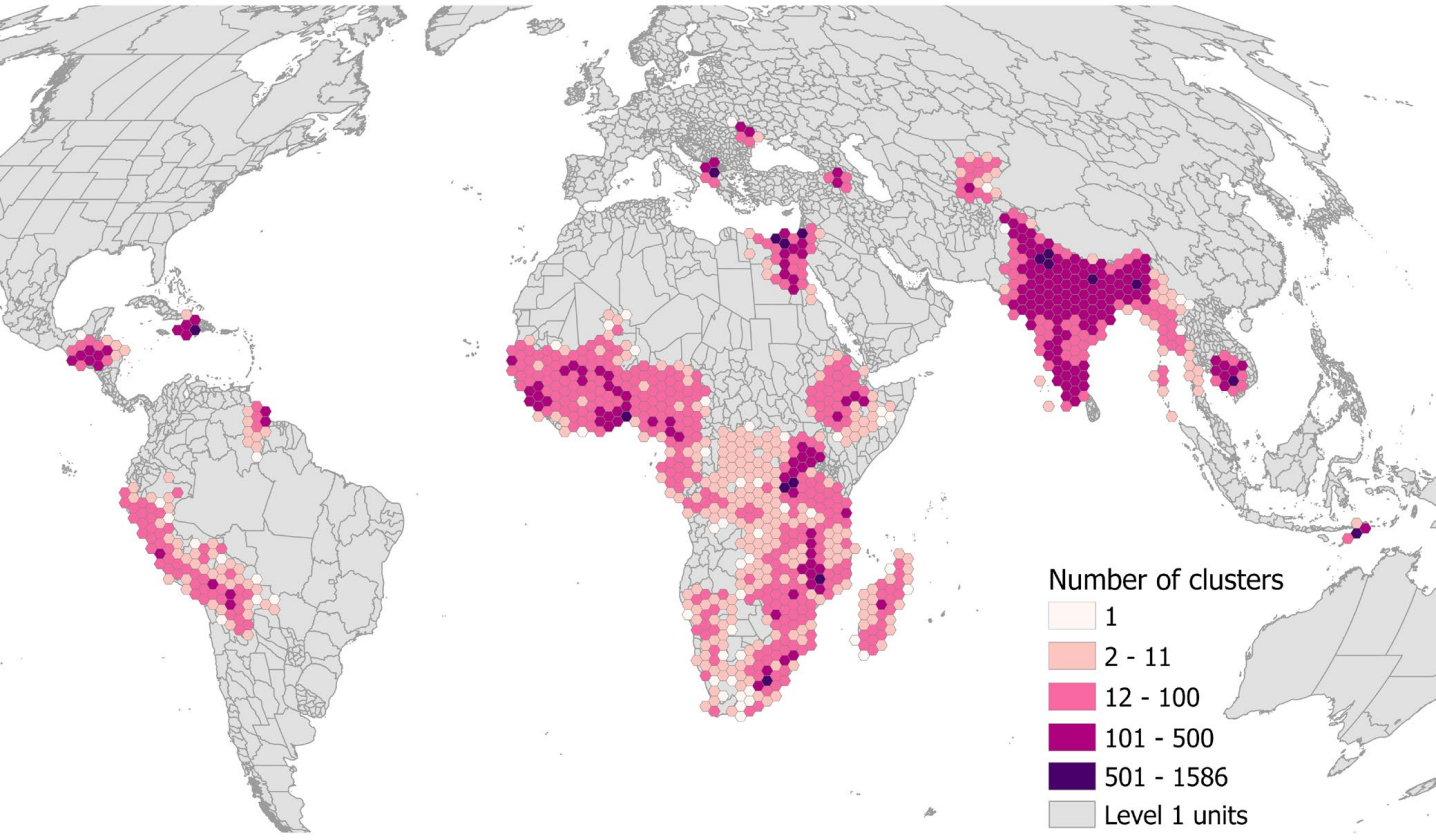
Density of DHS clusters across 46 countries (87 surveys) overlaid on GADM level‐1 administrative units.

We integrate three data sources to enable our analysis. First, we use data from the DHS Program, which collects nationally representative data on the health and sociodemographic characteristics of reproductive‐age women in low‐ and middle‐income countries. This project builds on previous scholarship that combines DHS surveys with climate data to analyze health and social outcomes (Grace et al. 2015; Gray and Thiede 2024; Muttarak 2021; Randell et al. 2020). We compile data for approximately 1.3 million women aged 15 to 49 from 87 surveys across 46 countries, the largest sample with information on anemia and location available at the time of data compilation (Figure 1; Table A1). In each survey round, the DHS program uses a stratified two‐stage sampling frame whereby clusters and then households are selected. We use survey weights in all regression analyses.

Second, we rely on climate data from the CRU TS, which provides monthly temperature and precipitation data with a 0.5° spatial resolution since 1901. We extract CRU data from 1981 to 2020 for this study (Harris et al. 2020). GPS points of DHS clusters were used to extract spatial means of temperature and precipitation using a 10‐km buffer, reflecting the fact that DHS clusters are randomly offset from 2‐ to 10‐km to ensure anonymity (Perez‐Heydrich et al. 2013). These values were then transformed into measures of exposure over the 24 months before each DHS interview period as described below.

Finally, we extract the province‐level location of each cluster using level‐1 subnational boundary files provided by the Database of Global Administrative Areas (GADM) (GADM 2022). We used DHS cluster GPS points to match cluster IDs to level‐1 provinces, which were then merged to the analytical dataset.

### 2.2 Analytic Approach

Selected DHS surveys measure the concentration of hemoglobin, a protein within the red blood cell that is needed to carry oxygen to bodily tissues. One common way to measure anemia status and hemoglobin concentrations in population research, including in the DHS, is via capillary blood samples and a portable field device (Pullum et al. 2017). The DHS program adjusts this measurement for altitude, and we use these values to determine an individual's anemia status based on WHO guidelines (WHO 2024a).^1^ We drop observations based on whether the hemoglobin measurement was missing (either completely in the data or coded as missing by DHS) and whether the value was plausible. We exclude implausible hemoglobin values for non‐pregnant women which are below and above 4.0 and 18.0 g/dL, respectively (DHS 2018). We also drop implausible values for pregnant women by excluding values below and above 3.0 and 17.0 g/dL, respectively (Nankinga and Aguta 2019). The primary outcome variables are any anemia (0/1) and moderate‐to‐severe anemia (0/1) as defined in Table A2 (WHO 2011).

**FIGURE A1:**
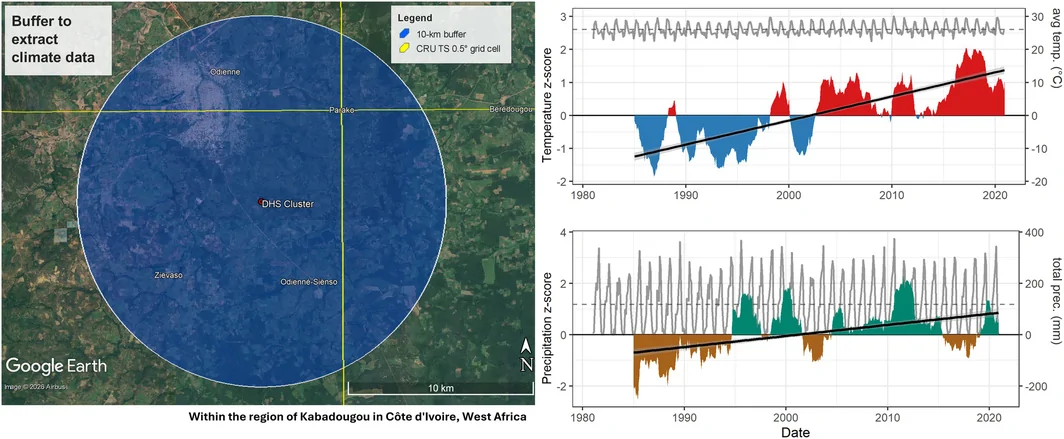
Location of the buffer and CRU grid cells alongside the time trend of climate values for one DHS cluster in Côte d'Ivoire.

Temperature and precipitation values were used to calculate 24‐month running means which were standardized to *z*‐scores (also known as standardized climate anomalies) using the local mean and standard deviation from the 1981–2020 climate history. Observing climate over 24 months allows us to consider longer exposures to adverse climate and lagged responses to climate shocks (Gray and Thiede 2024). Climate anomalies indicate the degree to which the climate deviates from the local, historical norm (Figure A1). These anomalies are on average uncorrelated with the historical climate and have increasingly been used to predict social outcomes (Call and Gray 2020; Gray and Wise 2016). These 24‐month temperature and precipitation anomalies were then attached to the individual‐level data using DHS cluster identifiers as well as the year and month of the survey interview. To allow for nonlinear and interactive effects, we also dichotomize these climate anomalies at less than −1 standard deviation or greater than 1 standard deviation to create measures of locally hot, cold, dry, and wet conditions, as well as combinations (or interactive categories) such as “hot‐and‐wet” or “cold‐and‐dry.” We likewise drop cases where there was no climate information associated with the DHS cluster, indicating DHS did not collect a GPS unit or the observations were not geocoded. After dropping observations based on hemoglobin and climate information, this resulted in the final analytic sample of 1 299 998 women of reproductive age.

This linked dataset is analyzed using binary logistic regression with fixed effects for provinces as well as region‐by‐time interactions. The model incorporates sampling weights as well as adjusts the standard errors for clustering. The estimating equation is:

$$\ln\left(\frac{p}{\left(1-p\right)}\right)=\alpha_{p}+\alpha_{r}t+{\mathrm{\delta C}}_{\mathrm{cm}}+\beta X_{\mathrm{ic}}$$

here $\alpha_{p}$ are province fixed effects, which control for constant characteristics within each province (e.g., baseline soil type). The term $\alpha_{r}t$ is an interaction between the world region *r* and the linear survey year *t*, accounting for region‐specific linear trends over time such as overall changes in anemia prevalence. World regions were defined as sub‐Saharan Africa (SSA), South and Southeast Asia (SEA), Latin America (LAT), and North Africa and Europe (NAE). ${\mathrm{\delta C}}_{\mathrm{cm}}$ represents the climate coefficients, δ, to be estimated and the observed climate anomalies for each cluster *c* and month in sequence *m*. $\beta X_{\mathrm{ic}}$ represents the column vector of coefficients, β, for controls as well as the row vector $X_{\mathrm{ic}}$ of control variables for individual *i* in cluster *c*. Controls used in this analysis are age, education, marital status, relationship to household head, number of children ever born, currently pregnant, any birth in past year, currently breastfeeding, current modern contraceptive use, and rural place of residence (Table A3). We selected these controls because of their relevance to human health and because they are not likely to be endogenous to recent climate exposures. We do not include wealth because it is not measured consistently across rounds and can be affected by the climate exposures of interest. We also control for the average historical temperature and precipitation and their standard deviations at the cluster level to account for sub‐provincial variations in the historical climate. We present results and report significance levels at *α* = 0.05 and note weakly associated relationships at the *α* = 0.1 level.

To provide insight into the mechanisms of climate effects on anemia, we also use parallel data collected by DHS on height and weight to define underweight as BMI ≤ 18.5 kg/m^2^ in a supplementary analysis. This supplementary analysis overlaps with some of the main results for Gray and Thiede's (2024) analysis, but we rely only on the sample of women where hemoglobin was collected. Likewise, we use binary logistic regression with fixed effects for this model. The motivation for this supplementary analysis is to observe how climate affects one of the major risk factors for anemia, malnutrition. If climate increases anemia risk as well as the risk of being underweight, then we have higher confidence in climate's acting on nutrition‐ or agriculture‐related mechanisms leading to anemia.

## 3 Results

Below we present descriptive statistics, results from the main regression analyses on the full sample, and results from regressions stratified by world region, residence, pregnancy status, and education. We also explore categorical and interactive climate measures, for the full sample and all stratifications. Finally, we present a supplementary analysis of BMI status to provide insight into potential mechanisms.

### 3.1 Descriptive Results

We begin by describing our sample. Sample sizes for provinces, clusters, and individuals are presented in Table A1. The largest national sample in the analytic dataset is from India. According to Table A3, 44.5% of all women were anemic with 23.1% in the moderate‐to‐severe category. The average hemoglobin level was 11.97 g/dL. Most women, 66.7%, resided in rural areas. Most women completed primary or secondary education at 22.6% and 41.3% of observations, respectively; 26.7% did not complete education and 9.4% completed higher education. Most women in the sample were married or living with a partner (68.9%). Around 11% of women had given birth in the past year, 6% were currently pregnant, and around 20% were breastfeeding. The average number of children ever born was 2.2. Approximately 29% of women used modern contraception. Across regions, anemia varied in prevalence (Table A4). For example, the prevalence of mild anemia ranged from 15.4% in LAT to 23.8% in SEA. The results for regression using only controls are presented in Table A5 (Models M1–M2).

### 3.2 Overall Climate Effects on Anemia

**FIGURE 2:**
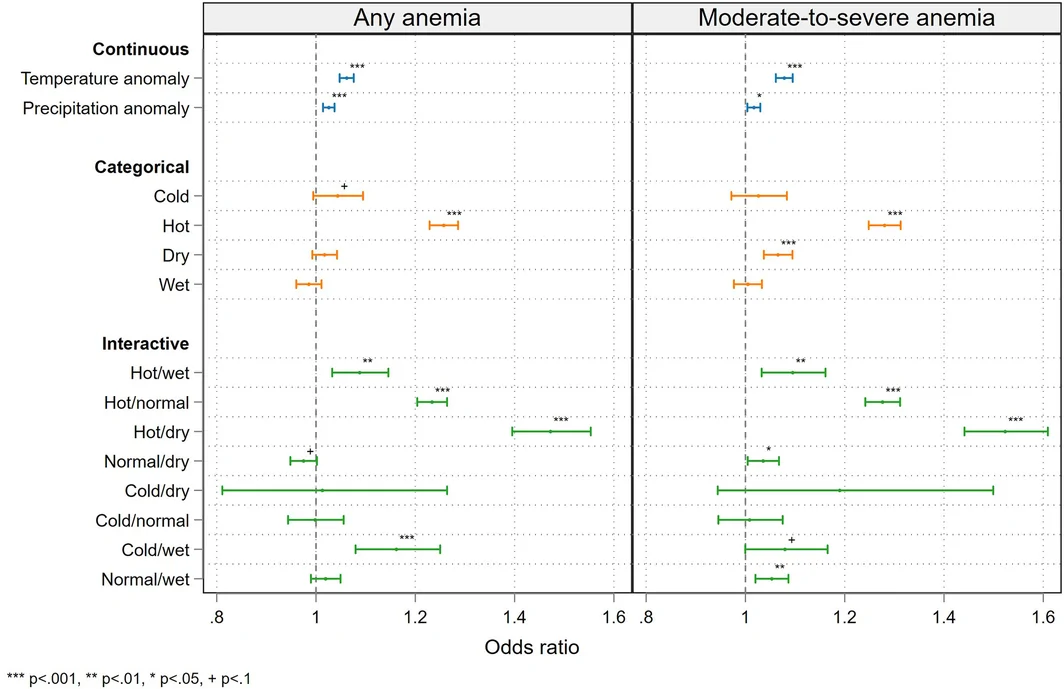
Odds ratios, confidence intervals, and significance tests for the effects of climate exposures on anemia using (1) continuous climate variables, (2) categorical climate variables, and (3) categorical and interactive climate variables (*N* = 1 299 997).

Our first analytic goal is to measure the associations between exposure to climate anomalies and anemia risk among women across the entire sample. We do so by fitting a series of regression models that respectively measure climate anomalies continuously (assuming monotonic impacts); categorically (allowing the impacts of above‐ and below‐average conditions to differ); and with categorical interactions (allowing temperature and precipitation categories to interact). We summarize results for six models. The first set of models (Figure 2, Table A6, models M3 and M4) contains results for continuous climate anomalies for the any anemia and moderate‐to‐severe anemia binary outcomes. The next set of models (Figure 2, Table A6, models M5 and M6) estimates the coefficients for categorical climate measures for the same binary outcomes. Lastly, the final set of models (Figure 2, Table A6, models M7 and M8) expresses results for combined interactive effects for any anemia and moderate‐to‐severe anemia binary outcomes.

For model M3, continuous temperature anomalies (OR = 1.06) and precipitation anomalies (OR = 1.03) significantly increase the odds of having any anemia. According to the point estimates, exposures to continuous temperature anomalies had a larger impact than continuous precipitation anomalies. A one unit increase in the temperature anomalies is associated with 1.06 times greater odds of being anemic than women exposed to average temperatures. A one unit increase in the precipitation anomaly, on the other hand, means the odds that a woman is anemic are 1.03 times greater than those not exposed. In model M4, temperature anomalies (OR = 1.08) and precipitation (OR = 1.02) also significantly increase the odds of moderate‐to‐severe anemia.

The models that include continuous measures of temperature and precipitation anomalies assume a monotonic association between climate exposures and anemia which may mask important nonlinearities. We account for such patterns by measuring temperature and precipitation categorically in models M5 and M6, while retaining the assumption that temperature and precipitation operate independently. In model M5, we find that hot periods (OR = 1.26) significantly increase any anemia risk, while cold periods have a weak positive association with any anemia (OR = 1.04, *p* < 0.1). In model M6, hot exposures likewise significantly increase odds of anemia for the moderate‐to‐severe outcome (OR = 1.28). Dry exposures contribute to moderate‐to‐severe anemia in model M6 (OR = 1.07). Exposures to cool spells in model M6 as well as periods of above‐average precipitation in models M5 and M6 are not associated with statistically significant changes in anemia risk.

We next combine temperature and precipitation as interactive conditions since, for example, the effects of hot spells may differ if they occur during periods when precipitation is much above or below average. In model M7, we find that hot‐and‐wet, hot‐and‐normal, and hot‐and‐dry conditions contributed to anemia in increasing order for any anemia. A similar pattern holds for these same exposures in model M8. Hot‐and‐dry conditions had the largest odds ratio for model M7 (OR = 1.47) and model M8 (OR = 1.52). The hot‐and‐wet effects were slightly weaker but still significant for model M7 (OR = 1.09) and model M8 (OR = 1.10). In model M7, the cold‐and‐wet exposure significantly increased any anemia (OR = 1.16). Likewise, in model M8, this same exposure was weakly but still significantly associated with more severe anemia outcomes (OR = 1.08, *p* < 0.1). Cold‐and‐dry and cold‐and‐normal exposures were not significantly associated with anemia in model M7 or in model M8. Normal‐and‐wet conditions were only associated with anemia in model M8 but not in model M7.

### 3.3 Tests for Heterogeneous Effects

**FIGURE 3:**
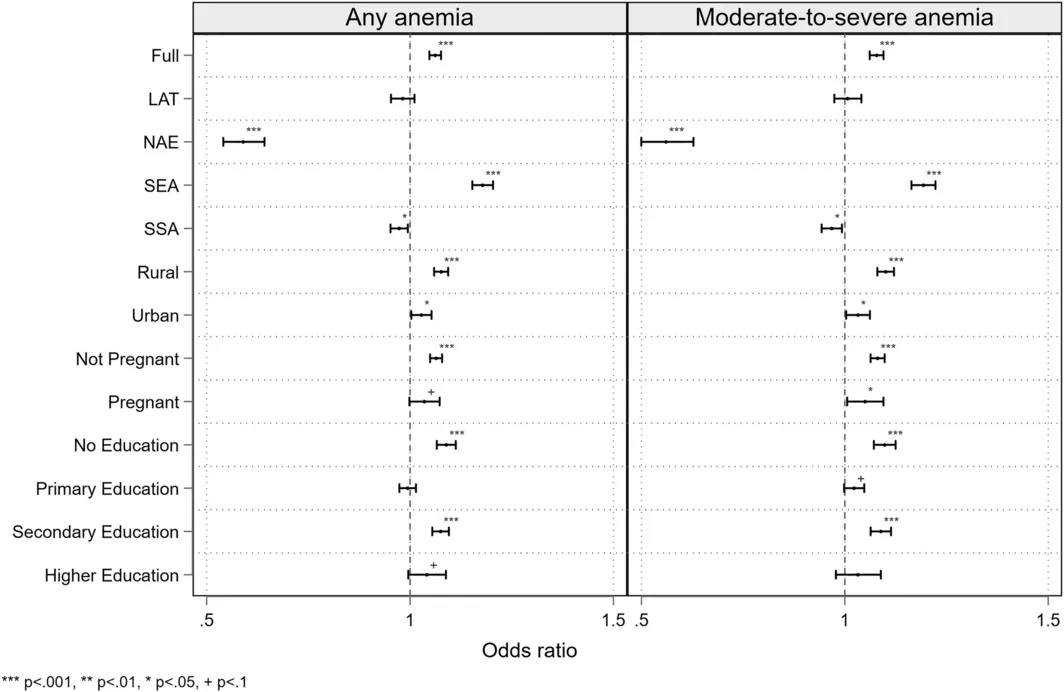
Odds ratios, confidence intervals, and significance tests for the effects of the continuous temperature anomalies stratified by region and subpopulation (*N* = 1 299 997).

**FIGURE 4:**
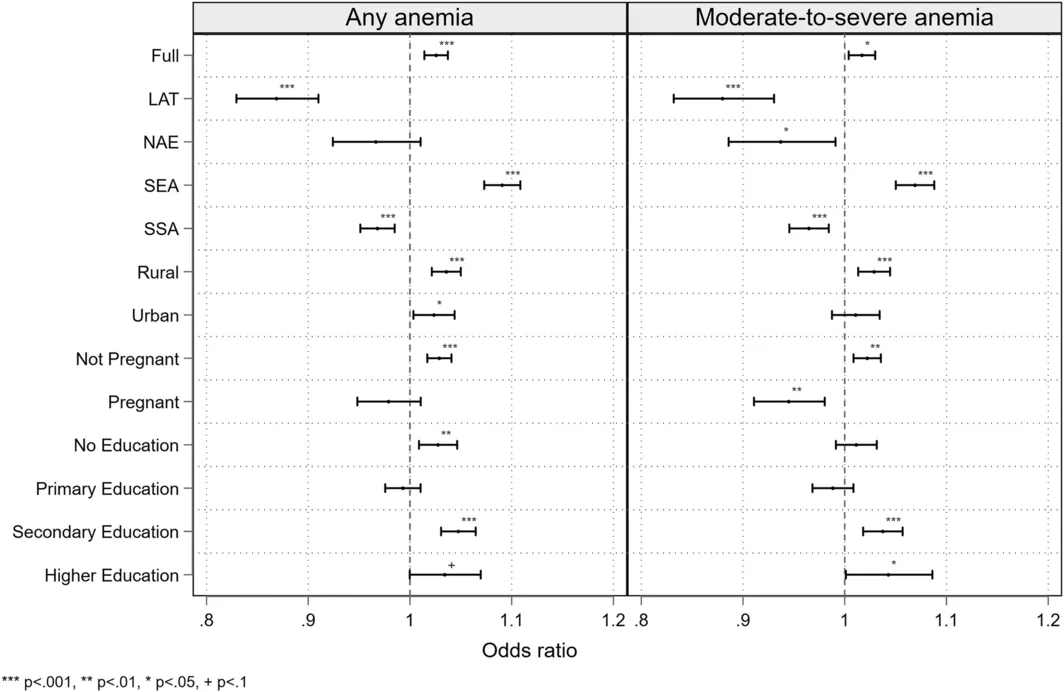
Odds ratios, confidence intervals, and significance tests for the effects of the continuous precipitation anomalies stratified by region and subpopulation (*N* = 1 299 997).

We next allow the associations between temperature and precipitation anomalies and anemia risk to vary across our large and diverse sample. We do so through a series of stratified regressions, the results of which are illustrated in Figures 3 and 4 (see also Table A7, models S1–S24). Here, the model with continuous climate anomalies was estimated for subsets of the data based on region, residence, pregnancy status, and education.

The regionally stratified models are fit for North Africa and Europe (NAE), Latin America and Caribbean (LAT), South and Southeast Asia (SEA), and sub‐Saharan Africa (SSA). We find that temperature significantly increases the risk for any anemia in SEA (OR = 1.18). On the other hand, temperature anomalies decreased any anemia risk in NAE (OR = 0.59) and SSA (OR = 0.97) with a similar pattern occurring for the moderate‐to‐severe outcome. There were no significant effects for temperature in the LAT region. Continuous precipitation anomalies increased any anemia risk for SEA (OR = 1.09) but decreased any anemia risk in LAT (OR = 0.87) and SSA (OR = 0.97), with the implication that precipitation deficits will increase anemia risk. A similar pattern exists for moderate‐to‐severe outcomes where precipitation decreases odds of anemia, and here we detect a significant effect in NAE (OR = 0.94).

From the rural–urban stratifications, we find that temperature increases the risk of any anemia in rural (OR = 1.08) and urban communities (OR = 1.03), and precipitation anomalies increase the risk of any anemia for rural (OR = 1.04) and urban communities (OR = 1.02). A similar pattern emerges for the moderate‐to‐severe outcome, but the effect of precipitation goes away for urban communities.

Temperature increases the risk of anemia in non‐pregnant women (OR = 1.06) as well as pregnant women (OR = 1.03, *p* < 0.1). A similar pattern exists for the moderate‐to‐severe outcome. Precipitation anomalies increase the risk of any anemia for non‐pregnant women (OR = 1.03) and the same is true for moderate‐to‐severe anemia (OR = 1.02). On the other hand, precipitation anomalies decrease the risk for moderate‐to‐severe anemia (OR = 0.94) for pregnant women while having no effect for the any anemia outcome.

When looking across educational attainment, temperature increases the risk for women with no education (OR = 1.09), women with secondary education (OR = 1.07), and women with higher education (OR = 1.04, *p* < 0.1). A similar pattern is observed with the moderate‐to‐severe outcome except for women in higher education (no significant effects) and women with primary education (OR = 1.02, *p* < 0.1). Continuous precipitation anomalies, meanwhile, increase the risk of any anemia for those women with no education (OR = 1.03), secondary education (OR = 1.05), as well as higher education (OR = 1.03, *p* < 0.1). A similar pattern emerges for the moderate‐to‐severe outcome except for women with no education, where the effect is non‐significant.

### 3.4 Supplementary Analyses

We analyzed stratified results for categorical and combined climate conditions to determine if they similarly impact anemia outcomes across strata or not. We refer readers to Figures A2, A3, A4, A5, A6, A7, A8, A9, A10, A11, A12, A13, Table A8 (models S25–S48), and Table A9 (models S49–S72). We observe heterogeneous results across strata with increased odds of anemia for hot, hot‐and‐normal, and hot‐and‐dry exposures in most but not all strata.

### 3.5 Climate and BMI

**FIGURE 5:**
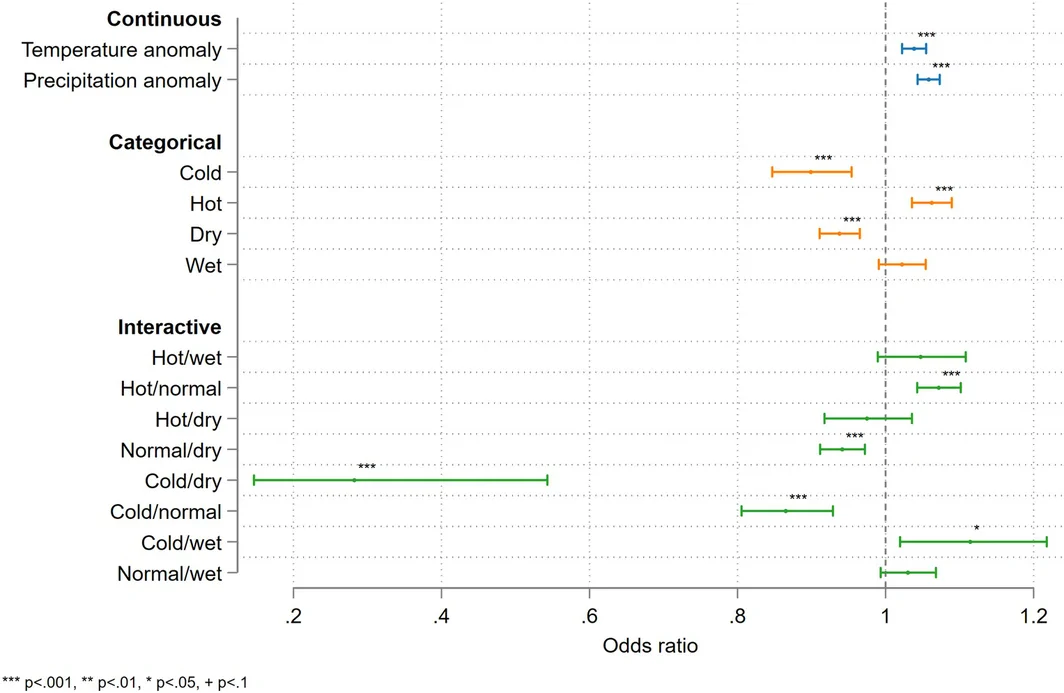
Odds ratios, confidence intervals, and significance tests for the effects of climate exposures on underweight status (BMI ≤ 18.5 kg/m^2^) (*N* = 1 277 689).

We examine climate's impact on underweight to examine if the impacts of climate on anemia may be driven by nutrition pathways (BMI ≤ 18.5 kg/m^2^; Figure 5 and Table A10, models S73–S75). The results are similar yet distinct from Gray and Thiede et al. (2024) insofar as the pattern of associations is comparable, but the sample is slightly smaller (i.e., we rely only on observations with hemoglobin measurements). We expect that if climate‐nutrition pathways are relevant to anemia, then climate will impact underweight outcomes similarly to anemia outcomes. Globally, continuous temperature anomalies (OR = 1.04) and precipitation anomalies (OR = 1.06) increase the odds ratio of the underweight outcome (Table A10, model S73). Hot exposures (Table A10, model S74) likewise increase underweight outcomes (OR = 1.06). In terms of interactive effects, hot‐and‐normal (OR = 1.07) and cold‐and‐wet (OR = 1.11) exposures increase underweight outcomes, which correspond to similar effects on anemia outcomes (Table A10, model S75). Conversely, dry exposures decrease the odds (OR = 0.94) of being underweight whereas this condition has a significant association with moderate‐to‐severe anemia. Hot‐and‐wet and hot‐and‐dry periods have no association with being underweight even though these conditions significantly increased anemia risk.

### 3.6 Continuous Hemoglobin Outcomes

Finally, we also replicate our main results using continuous hemoglobin outcomes for continuous, categorical, and interactive climate exposures (Table A11, models S76–S78). We find that the results of these models follow a similar pattern to the binary outcomes of our main analysis.

## 4 Discussion and Conclusion

This study reveals that climate anomalies increase anemia risk in a global sample of women of reproductive age. When measured across the entire sample, hot and dry extremes increase anemia risk, as do overlapping hot‐and‐dry, hot‐and‐normal, and hot‐and‐wet conditions. Hot‐and‐dry exposures are more strongly associated with anemia in SSA and SEA. Our results provide strong evidence of the threat from future climate change to the health of women of reproductive age, supporting mitigation efforts and future research. While certain climate conditions increase anemia risk at a global level, we also discover divergent trends across regions and stronger impacts in rural versus urban areas. Additionally, questions remain about mechanisms underlying these associations (Grace and Mikal 2019). Below, we highlight our core findings and speculate about plausible mechanisms based on existing literature.

Overall, our results are consistent with other studies analyzing the effects of temperature on health and nutrition in LMICs (Baker and Anttila‐Hughes 2020; Blom et al. 2022; Gray and Thiede 2024; Mora et al. 2017; Mueller and Gray 2018). For example, temperature anomalies increase the chances of being underweight in Chinese adults (Mueller and Gray 2018). Climate change is already reducing agricultural yields, possibly exacerbating malnutrition (Ray et al. 2019; Smith et al. 2017; Wheeler and von Braun 2013; Zhu et al. 2022). Zhu et al. (2023) previously identified a link between increasing temperatures and worse anemia outcomes for under‐5 children using DHS data from SSA. The authors suggest that hotter temperatures increase the risk for food insecurity, heat stress, and infectious diseases such as malaria.

We find that deviations from average precipitation levels result in worse health outcomes, which is also consistent with prior evidence. Previous studies have found that heavy rainfall in humid subtropical climates increases diarrheal diseases in children, while drought (or negative precipitation anomalies) does the same in savanna regions (Dimitrova et al. 2023). Droughts in India have exposed women to increased food insecurity, malnutrition, as well as water‐ and vector‐borne diseases (Algur et al. 2021; Sorensen, Saunik, et al. 2018). Moreover, higher temperature and heavy rainfall have been associated with diarrhea according to a recent review (Levy et al. 2016). In another study, Sindayihebura and Nkunzimana (2020) found that regions in Burundi that have experienced drought and decreased rainfall are the same regions associated with high levels of anemia in women.

Supplementary analyses reveal that climate does not impact BMI and anemia in the same ways. Hotter temperatures and above‐average precipitation both moderately increase underweight and anemia risk, but beyond this overlap the patterns begin to diverge. Dry spells reduce underweight risk but increase moderate‐to‐severe anemia risk, and hot‐and‐dry as well as hot‐and‐wet exposures have no association with low BMI while significantly increasing anemia. This suggests that climate may be uniquely affecting anemia pathways that do not contribute to low BMI. For example, hot‐and‐wet and hot‐and‐dry conditions may increase anemia but not BMI because iron‐rich foods such as animal products may be less available. Dry, hot‐and‐dry, and hot‐and‐wet conditions could alternatively be contributing to non‐dietary pathways to anemia, such as via infectious disease transmission.

**FIGURE A2:**
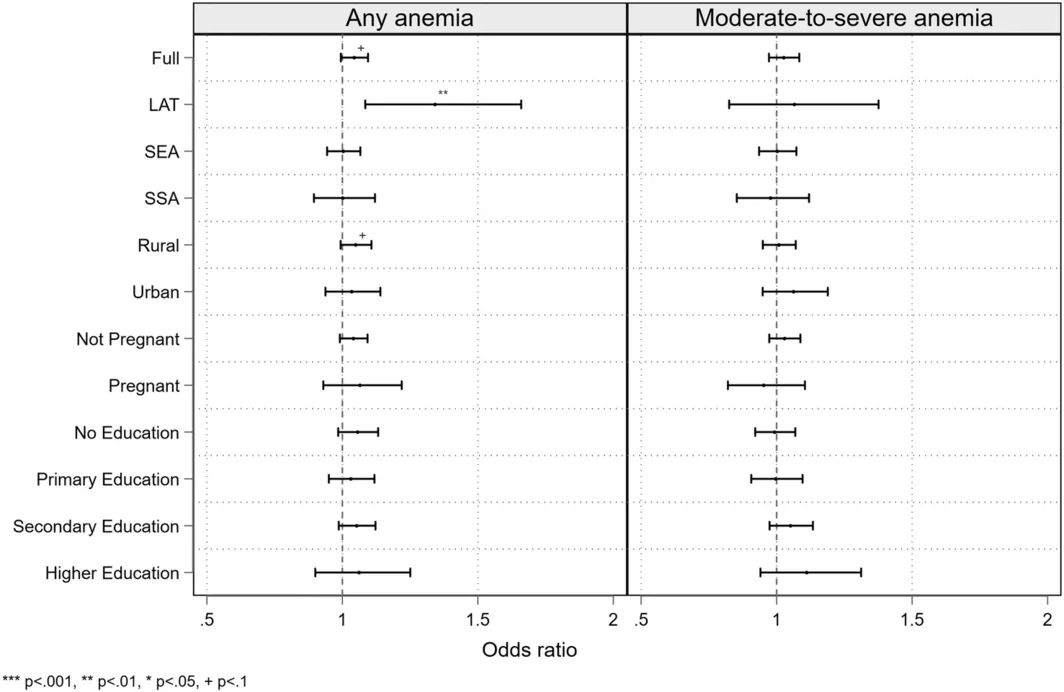
Odds ratios, confidence intervals, and significance tests for the effects of the categorical cold exposure stratified by region and subpopulation (*N* = 1 299 997).

**FIGURE A3:**
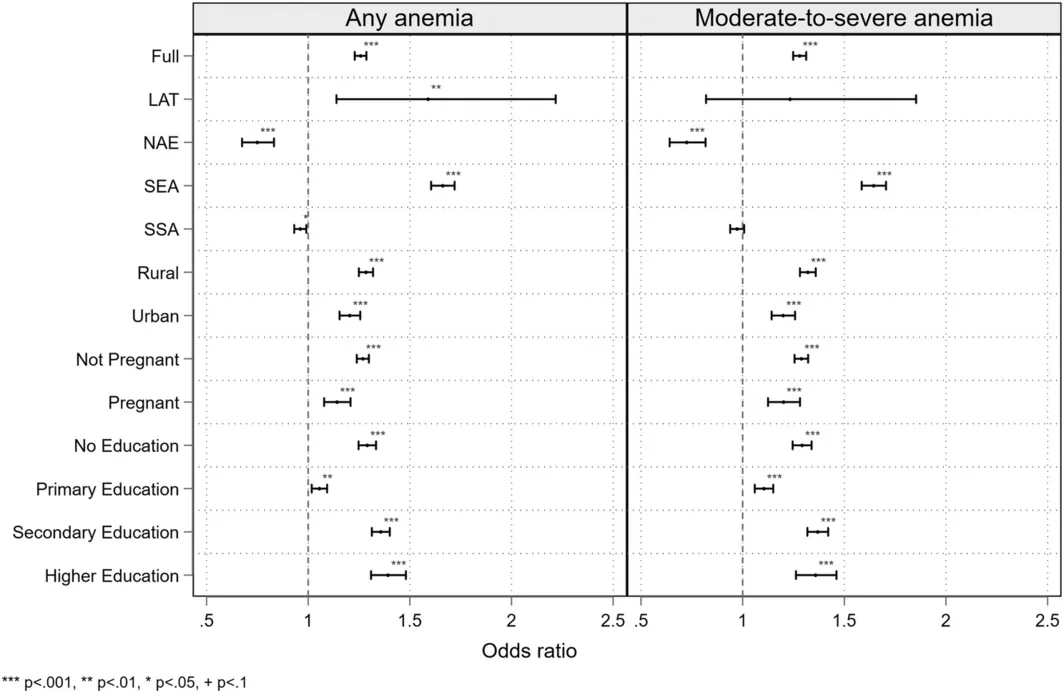
Odds ratios, confidence intervals, and significance tests for the effects of the categorical hot exposure stratified by region and subpopulation (*N* = 1 299 997).

**FIGURE A4:**
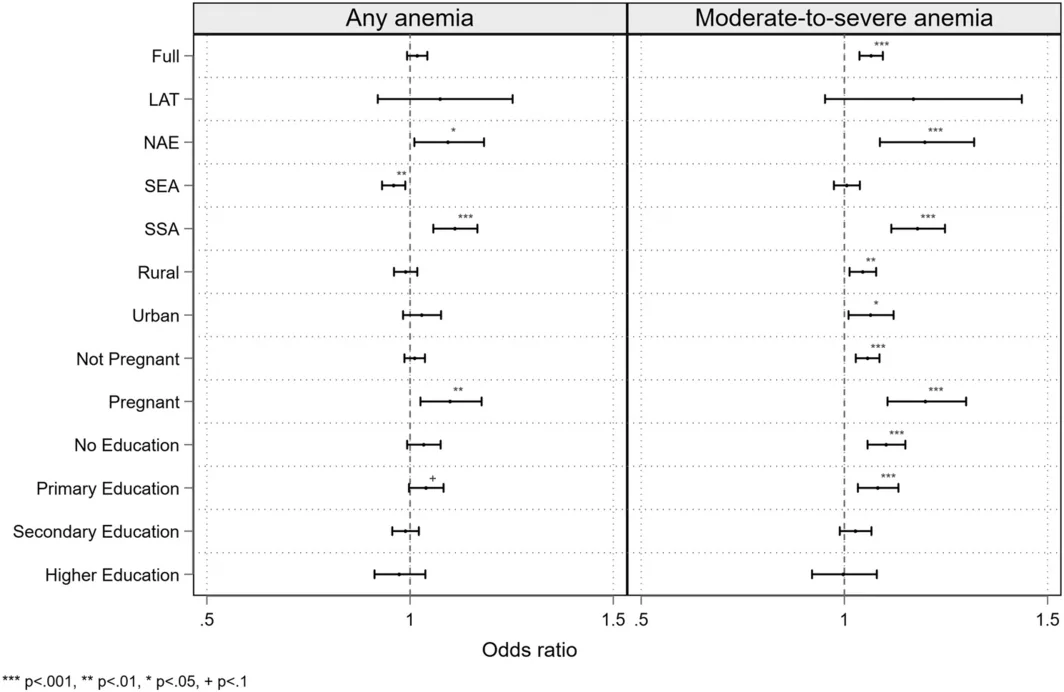
Odds ratios, confidence intervals, and significance tests for the effects of the categorical dry exposure stratified by region and subpopulation (*N* = 1 299 997).

**FIGURE A5:**
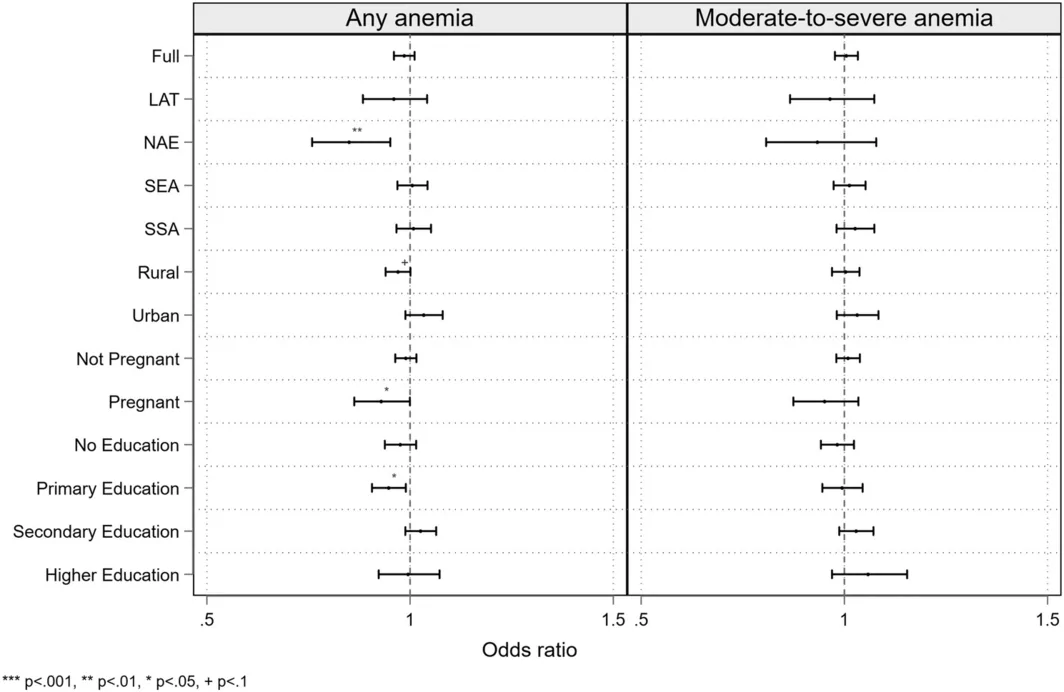
Odds ratios, confidence intervals, and significance tests for the effects of the categorical wet exposure stratified by region and subpopulation (*N* = 1 299 997).

**FIGURE A6:**
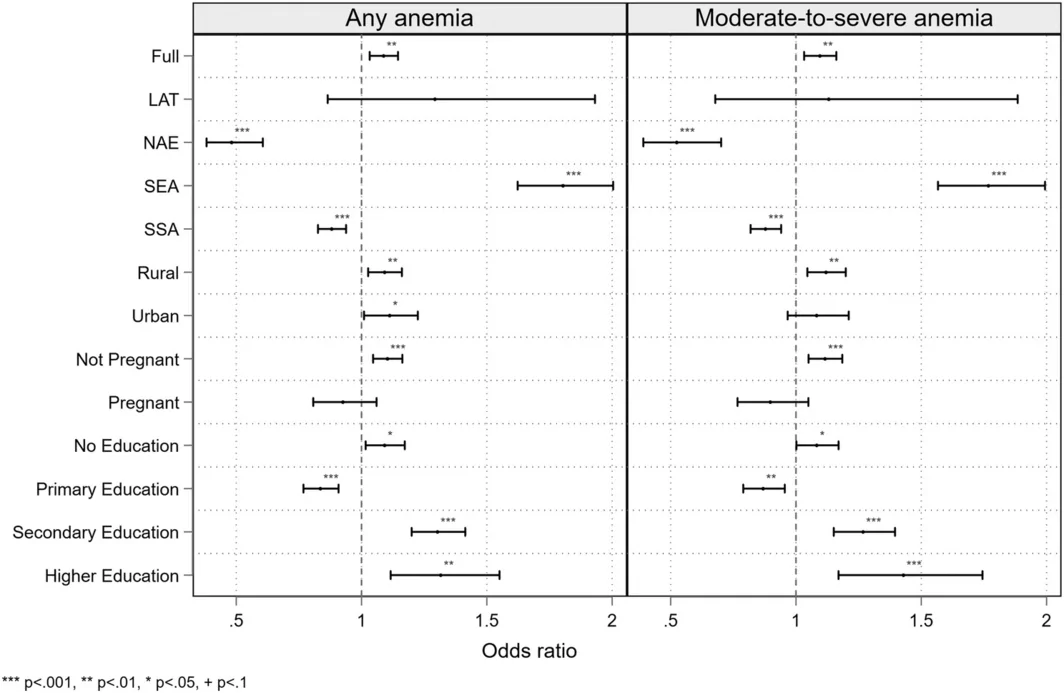
Odds ratios, confidence intervals, and significance tests for the effects of the interactive hot‐and‐wet exposure stratified by region and subpopulation (*N* = 1 299 997).

**FIGURE A7:**
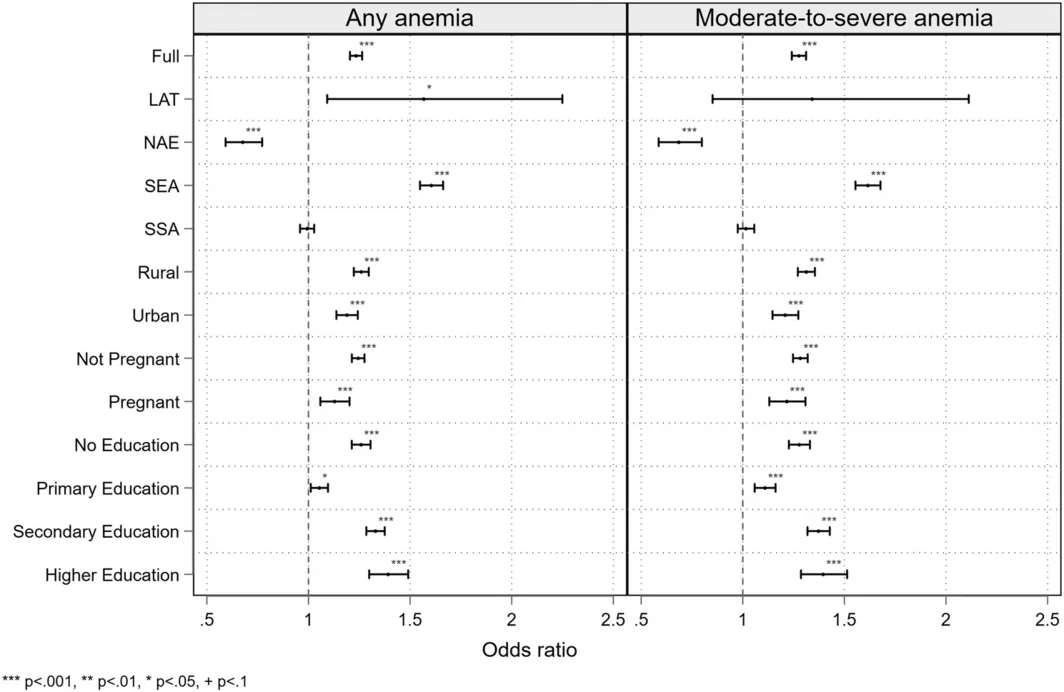
Odds ratios, confidence intervals, and significance tests for the effects of the interactive hot‐and‐normal exposure stratified by region and subpopulation (*N* = 1 299 997).

**FIGURE A8:**
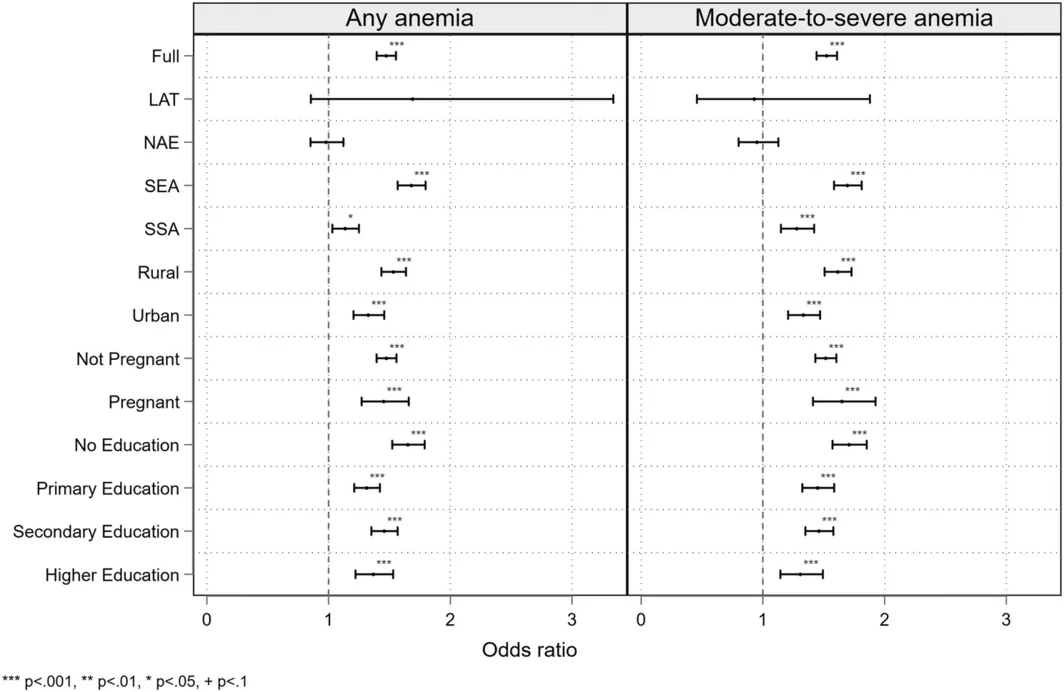
Odds ratios, confidence intervals, and significance tests for the effects of the interactive hot‐and‐dry exposure stratified by region and subpopulation (*N* = 1 299 997).

**FIGURE A9:**
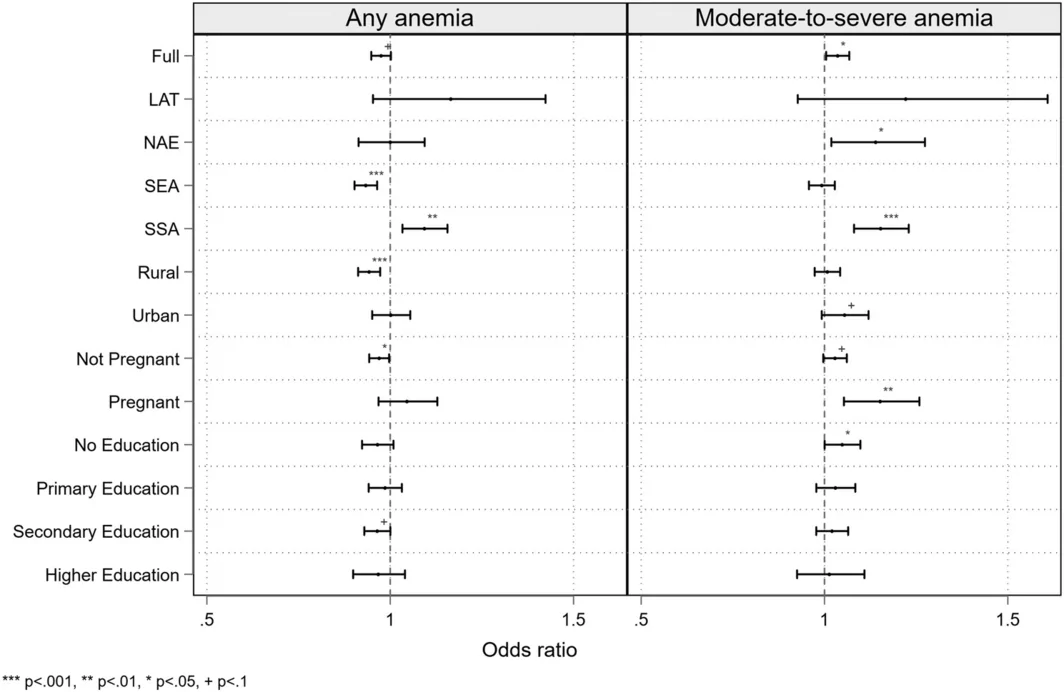
Odds ratios, confidence intervals, and significance tests for the effects of the interactive normal‐and‐dry exposure stratified by region and subpopulation (*N* = 1 299 997).

**FIGURE A10:**
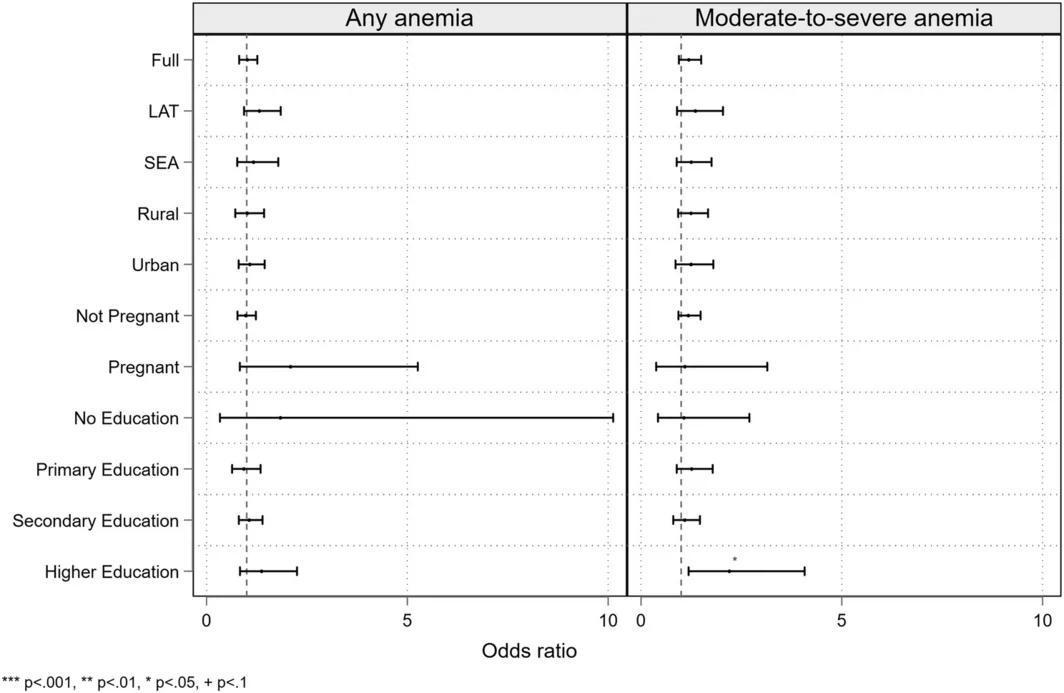
Odds ratios, confidence intervals, and significance tests for the effects of the interactive cold‐and‐dry exposure stratified by region and subpopulation (*N* = 1 299 997).

**FIGURE A11:**
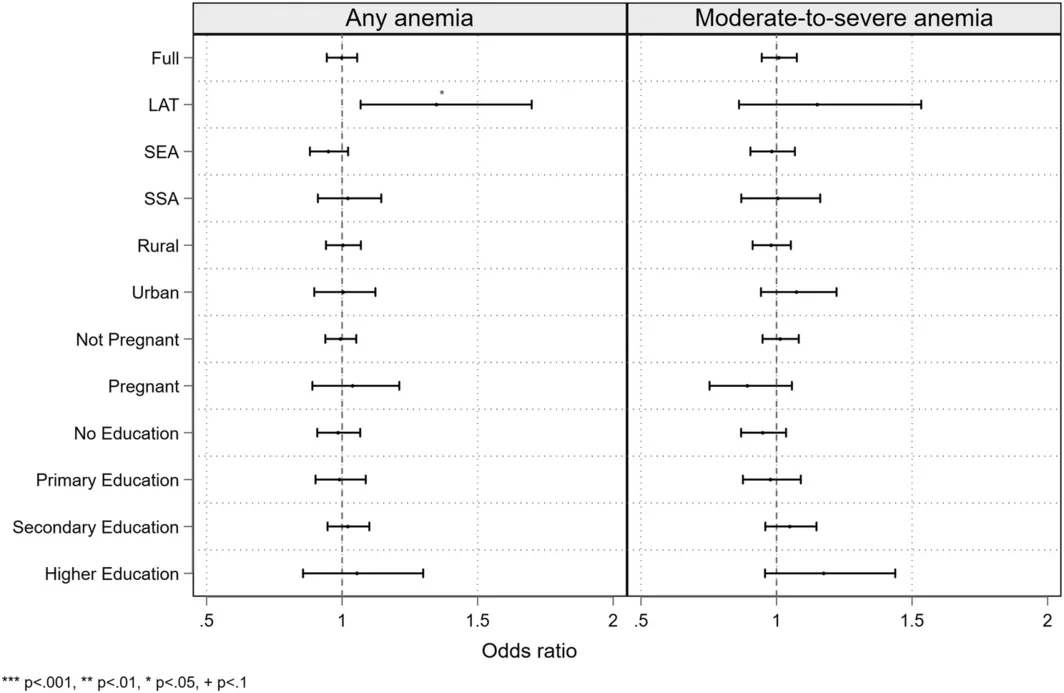
Odds ratios, confidence intervals, and significance tests for the effects of the interactive cold‐and‐normal exposure stratified by region and subpopulation (*N* = 1 299 997).

**FIGURE A12:**
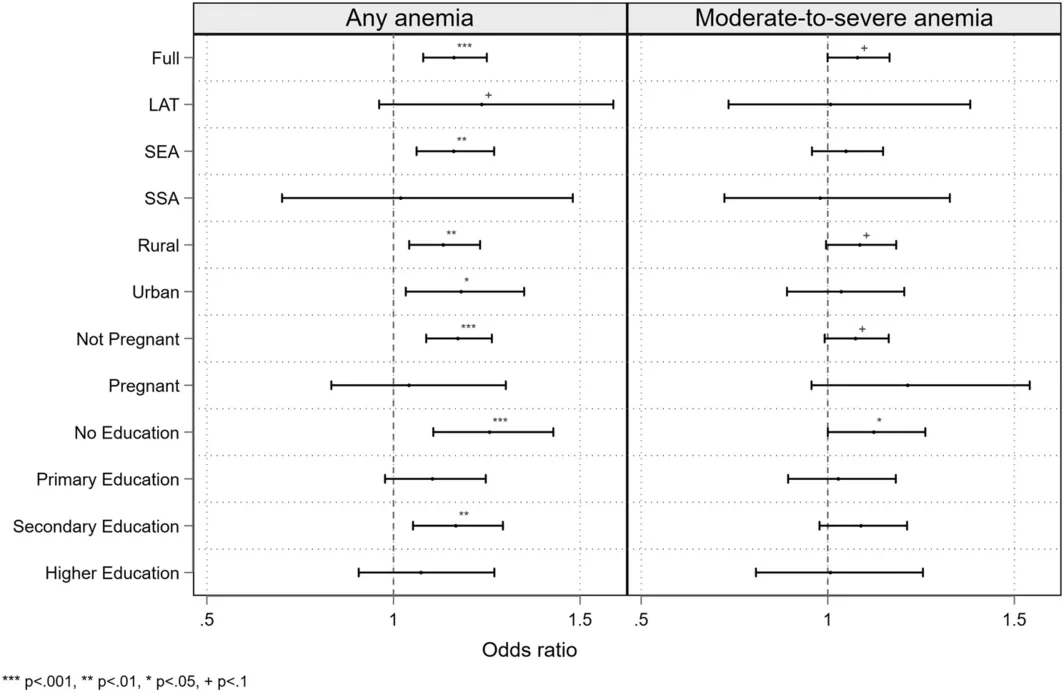
Odds ratios, confidence intervals, and significance tests for the effects of the interactive cold‐and‐wet exposure stratified by region and subpopulation (*N* = 1 299 997).

**FIGURE A13:**
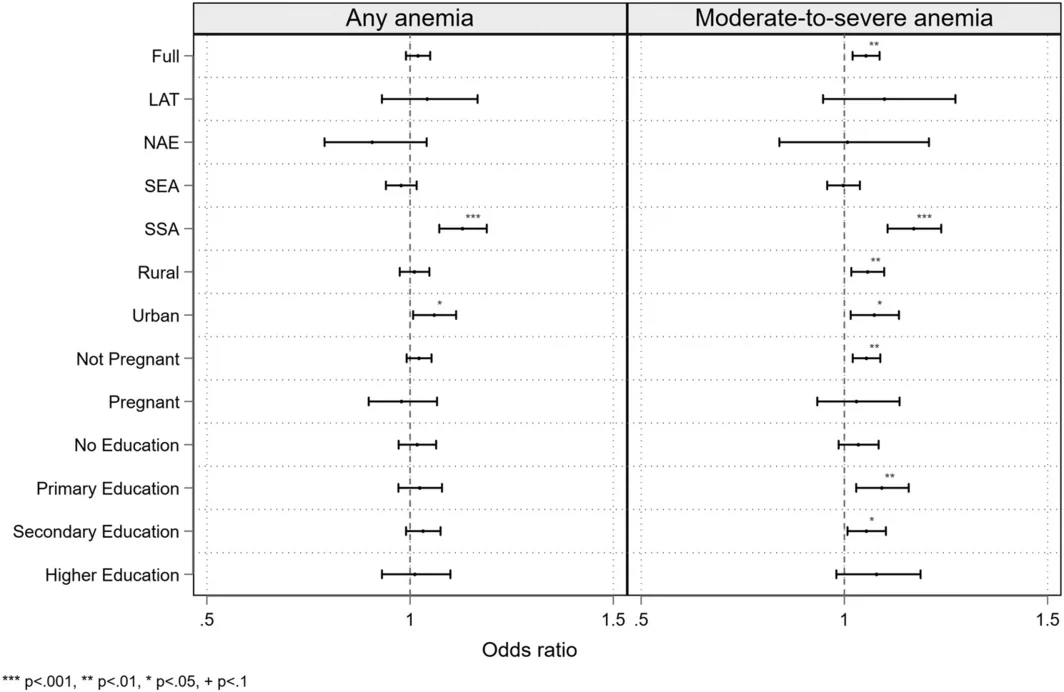
Odds ratios, confidence intervals, and significance tests for the effects of the interactive normal‐and‐wet exposure stratified by region and subpopulation (*N* = 1 299 997).

Temperature and precipitation conditions also had varying and divergent effects across world regions. Surprisingly, temperature anomalies significantly increased anemia odds in SEA but not in any other world region, and notable effects were also seen in hot, hot‐and‐wet, and hot‐and‐dry conditions in SEA. The relationship between higher temperature and negative health outcomes in SEA has been documented in other studies focusing on children as well as adult samples (Dimitrova et al. 2021; McMahon and Gray 2021). The association between precipitation deviations and health has likewise been reported in SEA, especially in studies on child health (Dimitrova and Muttarak 2020; Kumar et al. 2016; McMahon and Gray 2021). Positive precipitation anomalies and hot‐and‐wet conditions are consistent with extreme rainfall events, which could contribute to reduced agricultural yields and increased disease spread in the region. Finally, in SSA, dry and hot‐and‐dry spells increase anemia (Figures A2, A3, A4, A5, A6, A7, A8, A9, A10, A11, A12, A13 and A2, A3, A4, A5, A6, A7, A8, A9, A10, A11, A12, A13), which are consistent with drought conditions linked to food insecurity, malnutrition, or disease spread (Asmall et al. 2021). This aligns with other studies that suggest below‐average precipitation influences social and health conditions in SSA (Thiede et al. 2022).

Our results also show regional variation in the relationship between climate exposures and anemia. For example, when exposures are measured as continuous anomalies, positive temperature anomalies reduced anemia risk in NAE which is consistent with current climate‐health work studying women's anemia in Egypt and Jordan (Gemayel and Gray 2026). This work argues that hotter temperatures lead to dehydration, which potentially contributes to hemoconcentration in the blood thereby inflating hemoglobin values during point‐of‐care measurements. Another reason for this result in NAE could be idiosyncratic to the data itself as the subsample was smaller and geographically limited. Higher precipitation likewise decreases anemia risk in women in SSA and LAT. This pattern may be linked to agricultural pathways. For example, Ray et al. (2019) find that the impacts of climate change on agricultural production are not always negative and may, for the time being, improve yields in some areas of SSA and LAT. In Tanzania, more precipitation as well as warm‐and‐wet conditions improve household food security which is relevant for overall nutrition (Randell et al. 2022). Precipitation could also be an important mediator of increasing temperature's effects on health because of its importance to agriculture (Anttila‐Hughes et al. 2021). These divergent patterns are consistent with similar regional heterogeneity described in other quasi‐global analyses (Gray and Thiede 2024; Hoffmann et al. 2024; Randell and Gray 2019). Collectively, these findings demonstrate that the influences of climate are not uniform, but they do not provide a strong basis for explaining these differences. Further research should explore these trends at country and regional levels to unravel context‐specific mechanisms at work and improve predictions for where health burdens will increase or decrease under future climates.

Our work finds that continuous temperature and precipitation anomalies are more strongly associated with anemia in rural areas than urban areas. Other work analyzing the impacts of climate on a global sample of adult women finds that temperature threatens women's health in rural areas possibly linked to agriculture and agricultural labor pathways (Gray and Thiede 2024). However, this work and our findings also find that women in urban settings are also at risk to climate shocks, contrary to the increasingly discredited assumption that vulnerability is concentrated in rural areas.

Our findings, and the limitations to our study, provide motivation for continued research in this area. First, more work is needed to model the mechanisms linking climate exposures and anemia risk. The DHS data that we use have many strengths, but the cross‐sectional design of the surveys imposes significant constraints on modeling mediation processes. Future research should leverage longitudinal data with more detailed information on food consumption and illness to identify which causal pathways are operating (Meyer 2023; Randell et al. 2022). In addition to strengthening our general ability to make causal claims, evidence of causal mechanisms can improve explanations of the regional differences that we detect.

Second and relatedly, future research should also use such panel data to understand whether estimates of climate effects on anemia are affected by migration‐ and (or) mortality‐related biases. For example, our estimates would be biased if climate exposures change migration patterns among women (Garip and Reed 2025; Thiede et al. 2024) and those affected women differ systematically in their anemia risk. It would therefore be useful to understand the likely direction and magnitude of migration (and mortality) biases, and perhaps to also understand how the links between climate and anemia are moderated by migration.

Third, our attention to anemia—and the robust links between climate exposures and anemia that we uncover—underline the need to study other more targeted nutritional and health outcomes. While there are many compelling reasons for existing literature's emphasis on measures of weight and height (e.g., their accessibility, correlation with many indicators of health), the resulting indicators can be affected by multiple and disparate mechanisms, which makes interpretation difficult (Wheeler and von Braun 2013). Research using more specific measures of food consumption, micronutrient deficiency, or physiological or psychological stress, for example, could provide new evidence about exactly what dimensions of health are most vulnerable to climatic changes (McLaughlin et al. 2023).

Finally, we use global standards to define anemia based on thresholds set by the World Health Organization (WHO 2011, 2024b). In doing so, we rely on “any anemia” and “moderate‐to‐severe anemia” status categories to inform our analyses. Not only do hemoglobin thresholds for anemia vary across gender, age, and life stages, but evolutionary, biological, and social forces play a crucial role in structuring hemoglobin levels and iron needs especially for women (Miller 2023). However, current standards advise against adjusting hemoglobin concentrations for genetic ancestry, race, or ethnicity due to “insufficient evidence to change current adjustments and the complexity of operationalization” (WHO 2024b). Determining whether sub‐population differences exist between moderate and severe thresholds could be an avenue of future research that could inform global‐level analyses.

Together, the results highlight the potential effects of future climate change on human health through decreased agricultural yield, worsened nutrition, and heightened infectious disease spread. Iron supplementation is one solution to help improve anemia, but further efforts are needed to mitigate malnutrition risk in vulnerable populations that rely on rain‐fed agriculture as a primary source of food and income.

## Author Contributions

All authors contributed to the conception, analysis, and writing of this paper.

## Funding

We thank the Carolina Population Center (CPC) for providing institutional support through the CPC Population Research Infrastructure Center grant (P2C HD050924). This project was also supported by R03 HD101859 and by the CPC NICHD‐NRSA Biosocial Research Training program (T32 HD091058). Thiede acknowledges the support of the Population Research Institute at The Pennsylvania State University, which is supported by a grant from the Eunice Kennedy Shriver National Institute of Child Health and Human Development (P2CHD041025).

## Ethics Statement

The Demographic and Health Surveys (DHS) data are available for download and do not require ethics approvals for download. We rely on this secondary data for our analysis.

## Conflicts of Interest

The authors declare no conflicts of interest.

## Data Availability

The underlying data that support these findings are publicly available from the Climatic Research Unit, GADM, and the Demographic and Health Surveys (DHS) Program. The terms of use of the DHS data prohibit redistribution, including of the linked dataset that we analyze here.

## Endnotes

^1^
The variable is named v456 in DHS and is adjusted for both altitude as well as smoking (if the latter was collected) as indicated in the DHS Guide to Statistics (https://dhsprogram.com/data/Guide‐to‐DHS‐Statistics/Anemia_Status.htm).

## Appendix A

**TABLE A1:** Demographic and Health Survey rounds included in the analysis.

Code	Country	Start	End	Clusters	Provinces	*N*	Any anemia	Mod‐sev	BMI ≤ 18.5
AL5	Albania	2008	2009	450	12	7466	1330	431	210
AL7	Albania	2017	2018	711	12	10 429	2398	819	397
AM7	Armenia	2015	2016	313	11	5804	786	203	212
BD6	Bangladesh	2011	2011	600	7	5666	2364	960	1351
BF4	Burkina Faso	2003	2003	397	13	4160	2217	1205	825
BF6	Burkina Faso	2010	2010	541	13	7913	3800	2167	1136
BJ4	Benin	2001	2001	247	12	3123	2009	1308	322
BJ6	Benin	2011	2012	745	12	5017	2048	943	314
BJ7	Benin	2017	2018	540	12	7784	4496	2494	770
BO5	Bolivia	2008	2008	987	9	5701	2104	994	88
BU6	Burundi	2010	2011	376	17	4501	791	309	650
BU7	Burundi	2016	2017	552	17	8507	3167	1589	1496
CD5	DR Congo	2007	2007	292	26	4511	2215	1203	705
CD6	DR Congo	2013	2014	492	26	8605	3497	1726	1190
CI6	Cote d'Ivoire	2011	2012	338	14	4551	2383	1292	343
CM4	Cameroon	2004	2004	462	10	5113	2258	1208	303
CM6	Cameroon	2011	2011	577	10	7808	3124	1527	518
EG4	Egypt	2000	2005	2281	27	13 605	4637	1837	70
EG6	Egypt	2014	2014	1739	25	7138	1703	446	16
ET4	Ethiopia	2005	2005	525	11	5868	1567	865	1506
ET6	Ethiopia	2010	2011	571	11	14 885	2941	1466	4162
ET7	Ethiopia	2016	2016	621	11	14 078	3713	1871	3321
GA6	Gabon	2012	2012	331	9	5443	3180	1835	387
GH4	Ghana	2003	2003	410	10	5249	2379	1125	485
GH5	Ghana	2008	2008	404	10	4666	2758	1715	389
GH6	Ghana	2014	2014	423	10	4652	1962	952	285
GN4	Guinea	2005	2005	291	1	3814	1986	1120	486
GN6	Guinea	2012	2012	300	1	4689	2298	1216	553
GN7	Guinea	2018	2018	400	1	5260	2363	1219	483
GU6	Guatemala	2014	2015	853	22	25 265	3707	1318	693
GY5	Guyana	2009	2009	312	10	4441	1611	751	384
HN6	Honduras	2011	2012	1101	18	20 772	3089	992	910
HT4	Haiti	2000	2000	313	10	4748	2594	1476	575
HT5	Haiti	2005	2006	332	10	5112	2218	1243	732
HT6	Haiti	2012	2012	437	10	9200	4442	2225	1147
HT7	Haiti	2016	2017	450	10	9504	4555	2449	998
IA6	India	2015	2016	28 330	36	679 674	349 835	186 609	149 085
JO4	Jordan	2002	2002	164	12	1805	504	223	35
JO5	Jordan	2007	2007	463	12	5101	1750	743	61
JO6	Jordan	2012	2012	805	12	6950	2657	1287	112
JO7	Jordan	2017	2018	969	12	7110	3183	1531	79
KH4	Cambodia	2000	2000	470	24	3643	2136	1190	746
KH5	Cambodia	2005	2011	1155	25	17 182	7918	3709	3190
KH6	Cambodia	2014	2014	611	25	11 390	4988	2061	1538
KY6	Kyrgyz Republic	2012	2012	314	9	7987	2755	1402	512
LS4	Lesotho	2004	2005	377	10	2880	865	452	147
LS5	Lesotho	2009	2010	395	10	3851	988	500	226
LS6	Lesotho	2014	2014	399	10	3344	863	427	144
MB4	Moldova	2005	2005	399	36	7050	1920	731	416
MD5	Madagascar	2008	2009	585	6	8131	2951	1317	1955
ML4	Mali	2001	2001	388	9	3671	2327	1480	419
ML5	Mali	2006	2006	404	9	4674	2723	1578	556
ML6	Mali	2012	2013	413	6	5155	2669	1465	540
ML7	Mali	2018	2018	328	9	4811	3049	1827	469
MM7	Myanmar	2015	2016	441	15	12512	5627	2611	1765
MW4	Malawi	2004	2005	498	28	2729	1178	616	212
MW5	Malawi	2010	2010	827	27	7216	2107	1016	592
MW7	Malawi	2015	2016	850	28	7961	2685	1260	514
MZ6	Mozambique	2011	2011	609	11	13 507	7083	3964	985
NG7	Nigeria	2018	2018	1357	37	14 406	8349	4391	1589
NM6	Namibia	2013	2013	545	13	4322	898	397	556
NP5	Nepal	2006	2006	260	5	10 660	3671	1633	2485
NP6	Nepal	2011	2011	289	5	6084	1964	840	1019
NP7	Nepal	2016	2017	383	5	6421	2608	1161	1059
PE4	Peru	2000	2000	1356	26	6136	1862	787	77
RW4	Rwanda	2005	2005	456	5	5565	1363	691	507
RW6	Rwanda	2010	2015	984	5	13 634	2453	1007	874
SL5	Sierra Leone	2008	2008	339	4	3332	1493	805	350
SL6	Sierra Leone	2013	2013	434	4	7847	3572	1861	669
SN4	Senegal	2005	2005	363	14	4248	2546	1527	724
SN6	Senegal	2010	2011	382	14	5449	2922	1577	1139
SZ5	Swaziland	2006	2007	270	4	4518	1339	641	139
TG6	Togo	2013	2014	330	5	4802	2210	1095	358
TJ7	Tajikistan	2017	2017	365	5	10 577	4349	2048	768
TL5	Timor‐Leste	2009	2010	451	13	4089	907	362	1064
TL7	Timor‐Leste	2016	2016	454	13	4256	884	339	1098
TZ5	Tanzania	2009	2010	458	30	9545	4118	2125	1108
TZ7	Tanzania	2015	2016	608	30	13 088	6080	3158	1196
UG4	Uganda	2000	2001	265	49	5700	1887	971	456
UG5	Uganda	2006	2006	336	57	2549	1054	519	305
UG6	Uganda	2011	2011	399	57	2617	611	252	305
UG7	Uganda	2016	2016	685	56	5930	1911	853	528
ZA7	South Africa	2016	2016	641	9	2970	941	516	114
ZM7	Zambia	2018	2019	535	10	12 960	3942	1840	0
ZW5	Zimbabwe	2005	2006	396	10	7786	2830	1413	674
ZW6	Zimbabwe	2010	2011	393	10	7869	2337	1209	553
ZW7	Zimbabwe	2015	2015	400	10	9256	2524	1234	561

**TABLE A2:** Anemia level cutoffs for women by pregnancy status (WHO 2011).

	Non‐pregnant women	Pregnant women
Anemia categories	Hb (g/dL)	Hb (g/dL)
Any anemia	< 12.0	< 11.0
Mild	11.0–11.9	10.0–10.9
Moderate	8.0–10.9	7.0–9.9
Severe	< 8.0	< 7.0

**TABLE A3:** Summary statistics for the analytical sample.

Variable	Mean	Std. dev.	Min	Max
Hemoglobin (g/dL)	11.965	1.690	3	18
Any anemia	0.445	0.497	0	1
Moderate‐to‐severe	0.231	0.421	0	1
Age group				
15–19	0.189	0.391	0	1
20–24	0.177	0.382	0	1
25–29	0.166	0.372	0	1
30–34	0.140	0.347	0	1
35–39	0.126	0.332	0	1
40–44	0.106	0.308	0	1
45–49	0.097	0.295	0	1
Education				
None	0.267	0.442	0	1
Primary	0.226	0.419	0	1
Secondary	0.413	0.492	0	1
Higher	0.094	0.292	0	1
Missing	0.000	0.002	0	1
Marital Status				
Never married	0.249	0.433	0	1
Married/with partner	0.689	0.463	0	1
Widowed/etc.	0.062	0.241	0	1
Missing	0.000	0.001	0	1
Relation to head				
Head	0.089	0.285	0	1
Wife	0.459	0.498	0	1
Daughter/daughter‐in‐law	0.356	0.479	0	1
Other	0.096	0.295	0	1
Don't know/missing	0.000	0.005	0	1
Birth within 1 year	0.110	0.313	0	1
Children ever born	2.220	2.241	0	19
Pregnant	0.062	0.241	0	1
Currently breastfeeding	0.196	0.397	0	1
Using modern contraception	0.288	0.453	0	1
Rural	0.667	0.471	0	1
Interview year	2013.451	3.984	2000	2019
World region				
Latin America	0.070	0.255	0	1
North Africa/Europe	0.064	0.245	0	1
South and Southeast Asia	0.592	0.491	0	1
Sub‐Saharan Africa	0.274	0.446	0	1
Temperature anomalies	0.400	0.816	−2.208	2.552
Precipitation anomalies	0.103	0.979	−3.065	3.495
Categories				
Cold	0.033	0.178	0	1
Normal (temperature)	0.699	0.458	0	1
Hot	0.268	0.443	0	1
Dry	0.151	0.358	0	1
Normal (precipitation)	0.664	0.472	0	1
Wet	0.185	0.389	0	1
Interactions				
Normal	0.432	0.495	0	1
Hot/wet	0.031	0.172	0	1
Hot/normal	0.208	0.406	0	1
Hot/dry	0.029	0.168	0	1
Normal/dry	0.121	0.326	0	1
Cold/dry	0.001	0.027	0	1
Cold/normal	0.023	0.151	0	1
Cold/wet	0.009	0.092	0	1
Normal/wet	0.146	0.353	0	1
Temperature	23.610	4.787	−5.175	30.989
Precipitation	102.423	54.656	0.004	397.879
Mean temperature (24mo)	23.464	4.836	−5.449	30.493
SD temperature (24mo)	0.338	0.104	0.019	0.806
Mean precipitation (24mo)	101.671	53.869	0.012	413.078
SD precipitation (24mo)	10.772	5.594	0.005	57.576
Sample weight	0.998	0.942	0.001	44.811
*N*	1 299 998	—	—	—

**TABLE A4:** Anemia prevalence by world region and severity (*N* = 1 299 998).

Region	Severe	Moderate	Mild	None	Total
Latin America (LAT)	1.33%	12.13%	15.35%	71.19%	100.00%
North Africa and Europe (NAE)	0.69%	11.71%	17.97%	69.63%	100.00%
South and Southeast Asia (SEA)	2.14%	24.22%	23.75%	49.89%	100.00%
Sub‐Saharan Africa (SSA)	1.76%	19.09%	18.98%	60.16%	100.00%
Global	1.89%	21.17%	21.49%	55.46%	100.00%

**TABLE A5:** Odds ratios, standard errors, and significance tests for models M1 and M2 including only control variables.

Variables	Any anemia	Moderate‐to‐severe
	Model M1	Model M2
Age group		
20–24	0.968***	1.027*
	(0.009)	(0.012)
25–29	0.927***	0.997
	(0.011)	(0.013)
30–34	0.915***	0.988
	(0.011)	(0.015)
35–39	0.961**	1.043**
	(0.013)	(0.017)
40–44	0.972+	1.091***
	(0.014)	(0.019)
45–49	0.918***	1.004
	(0.014)	(0.018)
Education levels		
Primary	0.921***	0.914***
	(0.007)	(0.008)
Secondary	0.869***	0.852***
	(0.007)	(0.008)
Higher	0.766***	0.725***
	(0.010)	(0.011)
Missing	1.520	0.549
	(1.171)	(0.485)
Marital status		
Married/live with partner	0.970**	0.959***
	(0.010)	(0.012)
Widowed/etc.	1.121***	1.106***
	(0.016)	(0.019)
Relationship to head		
Wife	1.039***	1.029*
	(0.011)	(0.013)
Daughter/in‐law	1.058***	1.030*
	(0.012)	(0.013)
Other	1.070***	1.036*
	(0.014)	(0.016)
Don't know or missing	0.615	1.285
	(0.257)	(0.562)
Pregnant	1.105***	1.066***
	(0.013)	(0.014)
Any birth in past year	1.095***	1.118***
	(0.011)	(0.013)
# children ever born	1.019***	1.014***
	(0.002)	(0.002)
Currently breastfeeding	1.097***	1.035***
	(0.010)	(0.011)
Modern contraception	0.910***	0.888***
	(0.006)	(0.007)
Rural	1.055***	1.055***
	(0.010)	(0.011)
Mean temperature (24mo)	1.028***	1.013***
	(0.004)	(0.004)
SD temperature (24mo)	0.605**	0.944
	(0.103)	(0.174)
Mean precipitation (24mo)	1.002***	1.002***
	(0.000)	(0.000)
SD precipitation (24mo)	0.996+	0.994**
	(0.002)	(0.002)
Interview year	0.997	0.987*
	(0.004)	(0.006)
Region X interview year		
North Africa/Europe	1.019***	1.020**
	(0.005)	(0.007)
South and Southeast Asia	0.998	0.988+
	(0.005)	(0.007)
Sub‐Saharan Africa	0.993+	0.998
	(0.004)	(0.006)
*N*	1 299 997	1 299 997

*Note:* ****p* < 0.001, ***p* < 0.01, **p* < 0.05, + *p* < 0.1. Province fixed effects included but not shown.

**TABLE A6:** Odds ratios, standard errors, and significance tests for the effects of climate exposures on anemia for models M3–M8 using (1) continuous climate variables, (2) categorical climate variables, and (3) categorical and interactive climate variables.

Independent variables	Any anemia	Moderate‐to‐severe
Anomalies	Model M3	Model M4
Temperature anomaly	1.062***	1.078***
	(0.007)	(0.009)
Precipitation anomaly	1.026***	1.017*
	(0.006)	(0.007)
Joint test (*χ* ^2^)	88.281*******	90.027*******
*N*	1 299 997	1 299 997

Categorical	Model M5	Model M6
Cold	1.043+	1.026
	(0.026)	(0.029)
Hot	1.257***	1.280***
	(0.015)	(0.016)
Dry	1.017	1.065***
	(0.013)	(0.015)
Wet	0.985	1.005
	(0.013)	(0.014)
Joint test (*χ* ^2^)	390.889*******	391.243*******
*N*	1 299 997	1 299 997

Interactive	Model M7	Model M8
Hot/wet	1.088**	1.095**
	(0.029)	(0.033)
Hot/normal	1.234***	1.276***
	(0.015)	(0.018)
Hot/dry	1.472***	1.523***
	(0.040)	(0.043)
Normal/dry	0.975+	1.036*
	(0.014)	(0.016)
Cold/dry	1.013	1.190
	(0.115)	(0.140)
Cold normal	0.998	1.008
	(0.029)	(0.033)
Cold/wet	1.162***	1.079+
	(0.043)	(0.042)
Normal/wet	1.019	1.053**
	(0.015)	(0.017)
Joint test (*χ* ^2^)	447.820*******	445.274*******
*N*	1 299 997	1 299 997

*Note:* ****p* < 0.001, ***p* < 0.01, **p* < 0.05, + *p* < 0.1. Controls, province fixed effects, and region‐by‐year fixed effects included but not shown.

**TABLE A7:** Odds ratios, standard errors, and significance tests for models S1–S24 estimating the effects of climate exposures on anemia using continuous climate variables on stratified models.

Full	Any anemia	Moderate‐to‐severe
	Model M3	Model M4
Temperature anomaly	1.062***	1.078***
	(0.007)	(0.009)
Precipitation anomaly	1.026***	1.017*
	(0.006)	(0.007)
Joint test (*χ* ^2^)	88.281***	90.027***
*N*	1 299 997	1 299 997

LAT	Model S1	Model S2
Temperature anomaly	0.982	1.01
	(0.015)	(0.017)
Precipitation anomaly	0.869***	0.880***
	(0.021)	(0.025)
Joint Test (*χ* ^2^)	37.059***	23.416***
*N*	90 878	90 878

NAE	Model S3	Model S4
Temperature anomaly	0.589***	0.560***
	(0.026)	(0.033)
Precipitation anomaly	0.966	0.937*
	(0.022)	(0.027)
Joint test (*χ* ^2^)	165.557***	112.712***
*N*	83 034	83 034

SEA	Model S5	Model S6
Temperature anomaly	1.178***	1.193***
	(0.013)	(0.015)
Precipitation anomaly	1.091***	1.069***
	(0.009)	(0.010)
Joint Test (χ^2^)	283.603***	218.441***
*N*	769 562	769 562

SSA	Model S7	Model S8
Temperature anomaly	0.973*	0.968*
	(0.011)	(0.013)
Precipitation anomaly	0.968***	0.965***
	(0.009)	(0.010)
Joint Test (*χ* ^2^)	19.648***	18.782***
*N*	356 519	356 519

Rural	Model S9	Model S10
Temperature anomaly	1.076***	1.100***
	(0.009)	(0.010)
Precipitation anomaly	1.036***	1.029***
	(0.007)	(0.008)
Joint test (*χ* ^2^)	95.932***	106.386***
*N*	867 184	866 848

Urban	Model S11	Model S12
Temperature anomaly	1.028*	1.032*
	(0.013)	(0.015)
Precipitation anomaly	1.023*	1.011
	(0.010)	(0.012)
Joint Test (χ^2^)	9.403**	5.355+
*N*	432 755	432 656

Not Pregnant	Model S13	Model S14
Temperature anomaly	1.064***	1.081***
	(0.008)	(0.009)
Precipitation anomaly	1.029***	1.022**
	(0.006)	(0.007)
Joint test (*χ* ^2^)	92.759	93.736
*N*	1 219 625	1 219 625

Pregnant	Model S15	Model S16
Temperature anomaly	1.035+	1.050*
	(0.019)	(0.023)
Precipitation anomaly	0.979	0.945**
	(0.016)	(0.018)
Joint test (*χ* ^2^)	5.640+	15.939***
*N*	80 274	79 736

No education	Model S17	Model S18
Temperature anomaly	1.089***	1.098***
	(0.012)	(0.014)
Precipitation anomaly	1.027**	1.011
	(0.010)	(0.010)
Joint test (*χ* ^2^)	63.557***	56.505***
*N*	347 217	346 860

Primary education	Model S19	Model S20
Temperature anomaly	0.994	1.023+
	(0.010)	(0.013)
Precipitation anomaly	0.993	0.988
	(0.009)	(0.010)
Joint test (*χ* ^2^)	0.902	5.001+
*N*	294 389	294 257

Secondary education	Model S21	Model S22
Temperature anomaly	1.075***	1.088***
	(0.010)	(0.013)
Precipitation anomaly	1.047***	1.037***
	(0.009)	(0.010)
Joint test (*χ* ^2^)	80.404***	62.642***
*N*	536 318	536 270

Higher education	Model S23	Model S24
Temperature anomaly	1.041+	1.032
	(0.024)	(0.028)
Precipitation anomaly	1.034+	1.043*
	(0.018)	(0.022)
Joint test (*χ* ^2^)	6.250*	4.903+
*N*	121 783	120 977

*Note:* ****p* < 0.001, ***p* < 0.01, **p* < 0.05, + *p* < 0.1. Controls, province fixed effects, and region‐by‐year fixed effects included but not shown.

**TABLE A8:** Odds ratios, standard errors, and significance tests for models S25–S48 estimating the effects of climate exposures on anemia using categorical climate variables on stratified models.

	Any anemia	Mod‐to‐severe	Any anemia	Mod‐to‐severe	Any anemia	Mod‐to‐severe
	Full		Rural		Primary education	
	Model M5	Model M6	Model S33	Model S34	Model S43	Model S44
Cold	1.043+	1.026	1.049+	1.008	1.031	0.997
	(0.026)	(0.029)	(0.029)	(0.031)	(0.043)	(0.048)
Hot	1.257***	1.280***	1.284***	1.320***	1.055**	1.104***
	(0.015)	(0.016)	(0.018)	(0.020)	(0.019)	(0.023)
Dry	1.017	1.065***	0.989	1.045**	1.039+	1.082***
	(0.013)	(0.015)	(0.015)	(0.017)	(0.022)	(0.025)
Wet	0.985	1.005	0.970+	1.003	0.947*	0.994
	(0.013)	(0.014)	(0.016)	(0.017)	(0.021)	(0.025)
Joint test (*χ* ^2^)	390.889***	391.243***	328.243***	353.783***	17.956**	34.202***
*N*	1 299 997	1 299 997	867 184	866 848	294 389	294 257

	LAT		Urban		Secondary education	
	Model S25	Model S26	Model S35	Model S36	Model S45	Model S46
Cold	1.342**	1.065	1.034	1.062	1.052	1.051
	(0.146)	(0.139)	(0.052)	(0.061)	(0.035)	(0.041)
Hot	1.589**	1.233	1.204***	1.199***	1.357***	1.369***
	(0.270)	(0.256)	(0.026)	(0.029)	(0.022)	(0.026)
Dry	1.074	1.170	1.028	1.064*	0.988	1.027
	(0.084)	(0.123)	(0.024)	(0.028)	(0.017)	(0.020)
Wet	0.960	0.964	1.033	1.031	1.025	1.029
	(0.040)	(0.053)	(0.023)	(0.026)	(0.019)	(0.021)
Joint test (*χ* ^2^)	16.557**	9.624*	76.766***	57.168***	342.969***	272.669***
*N*	90 878	90 878	432 755	432 656	536 318	536 270

	NAE		Not pregnant		Higher education	
	Model S27	Model S28	Model S37	Model S38	Model S47	Model S48
Cold	—	—	1.041	1.029	1.061	1.111
	—	—	(0.026)	(0.029)	(0.089)	(0.094)
Hot	0.749***	0.724***	1.268***	1.288***	1.392***	1.358***
	(0.040)	(0.045)	(0.015)	(0.017)	(0.044)	(0.051)
Dry	1.093*	1.198***	1.011	1.057***	0.973	0.997
	(0.044)	(0.059)	(0.013)	(0.015)	(0.032)	(0.041)
Wet	0.850**	0.933	0.989	1.009	0.995	1.058
	(0.049)	(0.069)	(0.013)	(0.015)	(0.038)	(0.047)
Joint test (*χ* ^2^)	42.035***	42.831***	396.368***	382.628***	113.637***	68.359***
*N*	83 034	83 034	1 219 625	1 219 625	121 783	120 977

	SEA		Pregnant	
	Model S29	Model S30	Model S39	Model S40
Cold	1.003	1.002	1.065	0.952
	(0.031)	(0.035)	(0.074)	(0.072)
Hot	1.661***	1.644***	1.141***	1.201***
	(0.029)	(0.030)	(0.033)	(0.040)
Dry	0.959**	1.006	1.098**	1.199***
	(0.015)	(0.016)	(0.038)	(0.049)
Wet	1.005	1.012	0.929*	0.951
	(0.019)	(0.020)	(0.035)	(0.041)
Joint test (*χ* ^2^)	825.870***	721.333***	31.447***	53.561***
*N*	769 562	769 562	80 274	79 736

	SSA		No education	
	Model S31	Model S32	Model S41	Model S42
Cold	1.001	0.977	1.056	0.992
	(0.057)	(0.068)	(0.038)	(0.038)
Hot	0.960*	0.972	1.290***	1.292***
	(0.015)	(0.017)	(0.022)	(0.024)
Dry	1.110***	1.180***	1.033	1.103***
	(0.028)	(0.034)	(0.021)	(0.024)
Wet	1.008	1.026	0.976	0.982
	(0.022)	(0.024)	(0.020)	(0.021)
Joint test (*χ* ^2^)	27.123***	38.446***	231.391***	219.952***
*N*	356 519	356 519	347 217	346 860

*Note:* ****p* < 0.001, ***p* < 0.01, **p* < 0.05, + *p* < 0.1. Controls, province fixed effects, and region‐by‐year fixed effects included but not shown.

**TABLE A9:** Odds ratios, standard errors, and significance tests for models S49–S72 estimating the effects of climate exposures on anemia using interactive climate variables for all stratifications.

	Any anemia	Mod‐to‐severe	Any anemia	Mod‐to‐severe	Any anemia	Mod‐to‐severe
	Full		Rural		Primary education	
	Model M7	Model M8	Model S57	Model S58	Model S67	Model S68
Hot/wet	1.088**	1.095**	1.092**	1.120**	0.835***	0.869**
	(0.029)	(0.033)	(0.034)	(0.039)	(0.036)	(0.042)
Hot/normal	1.234***	1.276***	1.259***	1.312***	1.053*	1.109***
	(0.015)	(0.018)	(0.019)	(0.021)	(0.022)	(0.026)
Hot/dry	1.472***	1.523***	1.531***	1.614***	1.312***	1.449***
	(0.040)	(0.043)	(0.052)	(0.056)	(0.054)	(0.067)
Normal/dry	0.975+	1.036*	0.942***	1.007	0.986	1.030
	(0.014)	(0.016)	(0.015)	(0.018)	(0.023)	(0.027)
Cold/dry	1.013	1.190	1.013	1.243	0.925	1.261
	(0.115)	(0.140)	(0.179)	(0.187)	(0.177)	(0.224)
Cold/normal	0.998	1.008	1.003	0.980	0.990	0.977
	(0.029)	(0.033)	(0.033)	(0.036)	(0.047)	(0.054)
Cold/wet	1.162***	1.079+	1.133**	1.085+	1.104	1.028
	(0.043)	(0.042)	(0.048)	(0.048)	(0.069)	(0.073)
Normal/wet	1.019	1.053**	1.010	1.057**	1.023	1.092**
	(0.015)	(0.017)	(0.019)	(0.021)	(0.027)	(0.033)
Joint test (*χ* ^2^)	447.820***	445.274***	385.603***	413.224***	82.622***	105.655***
*N*	1 299 997	1 299 997	867 184	866 848	294 389	294 257

	LAT		Urban		Secondary education	
	Model S49	Model S50	Model S59	Model S60	Model S69	Model S70
Hot/wet	1.293	1.130	1.112*	1.082	1.303***	1.267***
	(0.265)	(0.295)	(0.055)	(0.062)	(0.054)	(0.062)
Hot/normal	1.567*	1.341	1.189***	1.208***	1.329***	1.372***
	(0.289)	(0.311)	(0.027)	(0.032)	(0.023)	(0.028)
Hot/dry	1.689	0.928	1.325***	1.332***	1.455***	1.460***
	(0.587)	(0.334)	(0.064)	(0.067)	(0.055)	(0.059)
Normal/dry	1.165	1.221	1.001	1.054+	0.964+	1.020
	(0.119)	(0.172)	(0.026)	(0.033)	(0.018)	(0.022)
Cold/dry	1.313	1.353	1.076	1.244	1.058	1.087
	(0.229)	(0.284)	(0.163)	(0.235)	(0.149)	(0.167)
Cold/normal	1.347*	1.150	1.004	1.073	1.021	1.049
	(0.160)	(0.169)	(0.057)	(0.071)	(0.039)	(0.048)
Cold/wet	1.236+	1.007	1.181*	1.036	1.167**	1.089
	(0.158)	(0.162)	(0.081)	(0.080)	(0.061)	(0.060)
Normal/wet	1.042	1.098	1.059*	1.073*	1.032	1.054*
	(0.060)	(0.083)	(0.027)	(0.030)	(0.022)	(0.024)
Joint test (*χ* ^2^)	20.92**	17.14*	86.980***	66.533***	354.260***	283.876***
*N*	90 878	90 878	432 656	432 656	536 318	536 270

	NAE		Not pregnant		Higher education	
	Model S51	Model S52	Model S61	Model S62	Model S71	Model S72
Hot/wet	0.481***	0.524***	1.103***	1.116***	1.316**	1.429***
	(0.057)	(0.078)	(0.030)	(0.034)	(0.110)	(0.146)
Hot/normal	0.676***	0.684***	1.244***	1.283***	1.391***	1.395***
	(0.046)	(0.054)	(0.016)	(0.018)	(0.049)	(0.058)
Hot/dry	0.977	0.950	1.474***	1.515***	1.367***	1.307***
	(0.069)	(0.083)	(0.042)	(0.044)	(0.079)	(0.089)
Normal/dry	1.000	1.139*	0.970*	1.028+	0.967	1.013
	(0.046)	(0.065)	(0.014)	(0.016)	(0.036)	(0.047)
Cold/dry	—	—	0.972	1.176	1.369	2.198*
	—	—	(0.116)	(0.139)	(0.348)	(0.692)
Cold/normal	—	—	0.994	1.013	1.055	1.174
	—	—	(0.029)	(0.034)	(0.112)	(0.122)
Cold/wet	—	—	1.172***	1.074+	1.073	1.007
	—	—	(0.045)	(0.044)	(0.092)	(0.113)
Normal/wet	0.906	1.007	1.022	1.054**	1.012	1.079
	(0.064)	(0.093)	(0.016)	(0.017)	(0.043)	(0.053)
Joint test (*χ* ^2^)	59.530***	51.669***	449.12	426.46	117.130***	78.963***
N	83 034	83 034	1 219 625	1 219 625	121 783	120 977

	SEA		Pregnant	
	Model S53	Model S54	Model S63	Model S64
Hot/wet	1.804***	1.768***	0.925	0.897
	(0.097)	(0.109)	(0.064)	(0.072)
Hot/normal	1.605***	1.615***	1.128***	1.216***
	(0.029)	(0.031)	(0.037)	(0.045)
Hot/dry	1.678***	1.694***	1.452***	1.649***
	(0.058)	(0.058)	(0.098)	(0.131)
Normal/dry	0.933***	0.992	1.045	1.151**
	(0.016)	(0.018)	(0.041)	(0.052)
Cold/dry	1.168	1.250	2.088	1.091
	(0.253)	(0.217)	(0.985)	(0.590)
Cold/normal	0.949	0.982	1.038	0.892
	(0.036)	(0.042)	(0.081)	(0.077)
Cold/wet	1.161**	1.049	1.041	1.214
	(0.053)	(0.049)	(0.118)	(0.148)
Normal/wet	0.978	0.997	0.979	1.030
	(0.019)	(0.020)	(0.043)	(0.052)
Joint test (*χ* ^2^)	873.606***	733.080***	46.533***	76.484***
*N*	769 562	769 562	80 274	79 736

	SSA		No education	
	Model S55	Model S56	Model S65	Model S66
Hot/wet	0.881***	0.878***	1.092*	1.083*
	(0.029)	(0.031)	(0.040)	(0.043)
Hot/normal	0.993	1.015	1.258***	1.278***
	(0.018)	(0.021)	(0.023)	(0.026)
Hot/dry	1.135*	1.278***	1.651***	1.707***
	(0.056)	(0.070)	(0.068)	(0.072)
Normal/dry	1.093**	1.153***	0.965	1.048*
	(0.031)	(0.038)	(0.022)	(0.025)
Cold/dry	—	—	1.837	1.067
	—	—	(1.600)	(0.505)
Cold/normal	1.021	1.005	0.984	0.949
	(0.059)	(0.074)	(0.040)	(0.042)
Cold/wet	1.019	0.980	1.257***	1.123*
	(0.194)	(0.152)	(0.082)	(0.067)
Normal/wet	1.129***	1.170***	1.017	1.034
	(0.030)	(0.034)	(0.024)	(0.025)
Joint test (*χ* ^2^)	64.38***	93.13***	286.171***	268.819***
*N*	356 519	356 519	347 217	346 860

*Note:* ****p* < 0.001, ***p* < 0.01, **p* < 0.05, + *p* < 0.1. Controls, province fixed effects, and region‐by‐year fixed effects included but not shown.

**TABLE A10:** Odds ratios, standard errors, and significance tests for models S73–S75 estimating the effects of climate exposures on underweight (BMI ≤ 18.5 kg/m^2^) using (1) continuous climate variables, (2) categorical climate variables, and (3) categorical and interactive climate variables.

Variables	BMI ≤ 18.5
Continuous	Model S73
Temperature anomalies	1.038***
	(0.008)
Precipitation anomalies	1.058***
	(0.008)
Joint test (*χ* ^2^)	77.522***
*N*	1 277 689

Categorical	Model S74
Cold	0.899***
	(0.027)
Hot	1.062***
	(0.014)
Dry	0.938***
	(0.014)
Wet	1.022
	(0.016)
Joint test (*χ* ^2^)	57.367***
*N*	1 277 689

Interactive	Model S75
Hot/wet	1.047
	(0.030)
Hot/normal	1.072***
	(0.015)
Hot/dry	0.975
	(0.030)
Normal/dry	0.941***
	(0.015)
Cold/dry	0.282***
	(0.094)
Cold/normal	0.865***
	(0.031)
Cold/wet	1.114*
	(0.050)
Normal/wet	1.030
	(0.019)
Joint test (*χ* ^2^)	83.004***
*N*	1 277 689

*Note:* ****p* < 0.001, ***p* < 0.01, **p* < 0.05, + *p* < 0.1. Controls, province fixed effects, and region‐by‐year fixed effects included but not shown.

**TABLE A11:** Coefficients, standard errors, and significance tests for models S76–S78 estimating the effects of climate exposures on hemoglobin (g/dL) using (1) continuous climate variables, (2) categorical climate variables, and (3) categorical and interactive climate variables.

Variables	Hemoglobin (g/dL)
Continuous	Model S76
Temperature anomalies	−0.048***
	(0.006)
Precipitation anomalies	−0.013**
	(0.005)
Joint test (F)	35.783***
*N*	1 299 998

Categorical	Model S77
Cold	−0.033+
	(0.019)
Hot	−0.180***
	(0.010)
Dry	−0.043***
	(0.010)
Wet	−0.001
	(0.011)
Joint test (F)	86.305***
*N*	1 299 998

Interactive	Model S78
Hot/wet	−0.073**
	(0.021)
Hot/normal	−0.165***
	(0.011)
Hot/dry	−0.347***
	(0.023)
Normal/dry	−0.006
	(0.011)
Cold/dry	−0.107
	(0.083)
Cold/normal	−0.002
	(0.023)
Cold/wet	−0.112***
	(0.030)
Normal/wet	−0.035**
	(0.012)
Joint test (F)	51.321***
*N*	1 299 998

*Note:* ****p* < 0.001, ***p* < 0.01, **p* < 0.05, + *p* < 0.1. Controls, province fixed effects, and region‐by‐year fixed effects included but not shown.
